# Heterointerface Engineering of FeOOH@Ni_3_N Electrocatalysts for Industrially Compatible Alkaline Water Electrolysis

**DOI:** 10.1002/smll.202513136

**Published:** 2025-12-26

**Authors:** Maria S. Metaxa, Ioannis Vamvasakis, Gerasimos S. Armatas

**Affiliations:** ^1^ Department of Materials Science and Engineering University of Crete Heraklion Greece

**Keywords:** electrocatalysis, heterostructured catalysts, in situ spectroscopy, oxygen evolution reaction, oxyhydroxides, transition metal nitrides

## Abstract

The rational design of earth‐abundant electrocatalysts is pivotal for advancing alkaline water electrolysis toward sustainable hydrogen production. Here, we report a hierarchical FeOOH@Ni_3_N heterostructure comprising a redox‐active iron oxyhydroxide overlayer conformally coupled with a conductive trinickel nitride core directly grown on nickel foam. This hybrid catalyst drives the oxygen evolution reaction (OER) with ultralow overpotentials of 209, 245, and 284 mV at 10, 100, and 500 mA cm^−2^, respectively, while maintaining exceptional stability under industrial‐level operations. Integrated into a two‐electrode electrolyzer, FeOOH@Ni_3_N achieves current densities of 10, 500, and 1000 mA cm^−2^ at cell voltages of only 1.49, 1.72, and 1.78 V, outperforming noble‐metal‐based benchmarks. Operando/in‐situ spectroscopies, combined with electrokinetic and isotope‐effect analyses, reveal that enhanced intrinsic activity originates from reconstructed proton–electron transfer pathways at the Fe–Ni heterointerface. Strong interfacial coupling stabilizes high‐valent Ni^4+^ = O/,Fe^4+^ = O species and promotes an unconventional dual‐site hydroxyl nucleophilic attack mechanism, wherein OH^−^ attack on Fe^4+^ = O forms a bridging *OOH intermediate as the O─O bond‐forming step, synergistically assisted by adjacent Ni centers. These findings delineate a clear structure–activity–stability relationship for Fe–Ni heterostructures and showcase heterointerface engineering of conductive nitrides with oxyhydroxides as a scalable strategy for developing durable, high‐rate OER electrocatalysts.

## Graphical abstract

A hierarchically structured FeOOH@Ni_3_N electrocatalyst supported on Ni foam enables high‐rate alkaline water electrolysis with industrial‐level stability. Operando spectroscopy reveals that the Fe–Ni heterointerface stabilizes high‐valent Ni^4+^ = O/Fe^4+^ = O species and promotes a hydroxyl nucleophilic attack (HNA) mechanism, wherein OH^−^ attack on electrophilic Fe^4+^ = O forms a bridging Fe^3+^–OOH⋅⋅⋅O–Ni^3+/4+^ intermediate, facilitating efficient O─O bond formation and rapid OER kinetics.

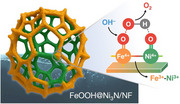

## 1 Introduction

The ever‐growing demands for clean and sustainable energy has intensified interest in hydrogen as a high‐density, carbon‐free energy carrier [1]. Among the available production routes, alkaline water electrolysis (AWE) powered by renewable electricity is one of the most attractive technologies for large‐scale hydrogen generation [2, 3]. Compared with proton‐exchange membrane (PEM) electrolysis, AWE offers key advantages, including the use of earth‐abundant catalysts, lower materials cost, and greater tolerance to impure water feeds [4]. However, the overall energy efficiency of AWE is limited by the sluggish oxygen evolution reaction (OER) at the anode [5] – a multistep four‐electron/four‐proton process (4OH^−^ → O_2_ + 2H_2_O + 4e^−^), involving O─H bond cleavage and O─O bond formation, which imposes large thermodynamic and kinetic barriers [6, 7, 8]. As a result, innovative strategies are urgently needed to greatly improve the efficiency of oxygen evolution electrocatalysts for expediting water‐alkali electrolysis at practically‐relevant current densities (>500 mA cm^−2^) with low overpotentials (<300 mV) and long‐term stability [9].

Noble‐metal oxides such as IrO_2_ and RuO_2_ are benchmarks for OER catalysts, especially at high current densities (>200 mA cm^−2^). Nevertheless, their high cost, scarcity, and inferior stability preclude large‐scale implementation [10]. This has motivated intensive exploration of earth‐abundant transition‐metal‐based catalysts, particularly Ni‐, Fe‐, and Co‐containing (oxy)hydroxides, which provide tunable electronic structures, abundant redox‐active centers, and rich populations of undercoordinated metal sites that favor OH^−^ adsorption and activation [11, 12, 13, 14, 15]. Despite notable progress, most non‐precious catalysts still demand high overpotentials and suffer from structural degradation under harsh alkaline conditions, where repeated redox cycling induces lattice strain, cracking, and dissolution of the oxide or (oxy)hydroxide framework. Therefore, strategies that can simultaneously enhance intrinsic catalytic activity and ensure structural robustness are essential. Recent advances in catalyst design have demonstrated several promising approaches, including heterostructure construction [16, 17, 18, 19, 20, 21], surface defect modulation [22, 23], and heteroatom doping [24, 25, 26, 27], to regulate charge transport and optimize catalytic performance. Among them, deliberate heterointerface engineering has emerged as an especially effective tactic to overcame the limitation of catalytic activity and improve the intrinsic activity of electrocatalysts. By coupling complementary materials, heterointerfaces can synergistically modulate the local electronic structure, optimize intermediate adsorption energies, and accelerate reaction kinetics [28, 29]. Importantly, many oxygen‐evolving catalysts, such as oxides, nitrides, sulfides, and phosphides, undergo electrochemical surface reconstruction during OER, generating dynamic MO*_x_* H*_y_* (M = Fe, Co, Ni) overlayers with abundant active sites and re‐equilibrated interfacial electronic states that enhance OH^−^ adsorption and catalytic turnover [30, 31, 32, 33]. However, the amorphous and evolving nature of these active surficial catalytic sites complicate mechanistic understanding and poses great challenges in establishing clear structure‐performance relationships.

Here, we present a hierarchical heterostructured electrocatalyst consisting of a conductive trinickel nitride (Ni_3_N) core and a redox‐active iron oxyhydroxide (*α*‐FeOOH) overlayer directly integrated on 3D nickel foam. Transition‐metal nitrides such as Ni_3_N provide metallic‐like conductivity, corrosion resistance, and emerging OER activity, making them ideal scaffolds for constructing hybrid systems [34, 35, 36]. Our catalyst is synthesized through a sequential nitridation‐impregnation‐oxidation strategy, yielding a compact *α*‐FeOOH shell that intimately integrates with Ni_3_N core. The *α*‐FeOOH overlayer introduces redox‐active Fe sites that not only enhance OH^−^ adsorption but also stabilize the in situ generated *γ*‐NiOOH phase. Operando/in‐situ Raman, ultraviolet‐visible, and electrochemical spectroscopies, complemented by electrokinetic analyses and isotope‐probing experiments, unveil that the Fe–Ni heterointerface fundamentally reconfigures proton‐electron transfer kinetics, stabilizes high‐valent Ni^4+^ = O/Fe^4+^ = O species, and establishes a cross‐interfacial route for oxygen intermediate activation. This dynamic dual‐site synergy disrupts the conventional adsorption‐energy scaling relationship among oxygenated intermediates on single‐site catalysts, thereby accelerating reaction turnover and intrinsically enhancing OER kinetics. As a result, the FeOOH@Ni_3_N/NF catalyst achieves ultralow overpotentials at high current densities, excellent long‐term stability, and high performance in a two‐electrode electrolyzer superior to noble‐metal‐based and industrial benchmarks. This work not only demonstrates a robust, earth‐abundant OER electrocatalyst with industrially‐relevant activity and durability but also provides molecular‐level mechanistic insights into dynamic active‐site evolution. Importantly, these findings unveil fundamental design principles for rational heterointerface engineering, paving the way toward scalable and cost‐effective catalysts for high‐rate water‐splitting electrolysis.

## 2 Results and Discussion

### 2.1 Electrocatalysts Synthesis and Structural Characterization

**FIGURE 1:**
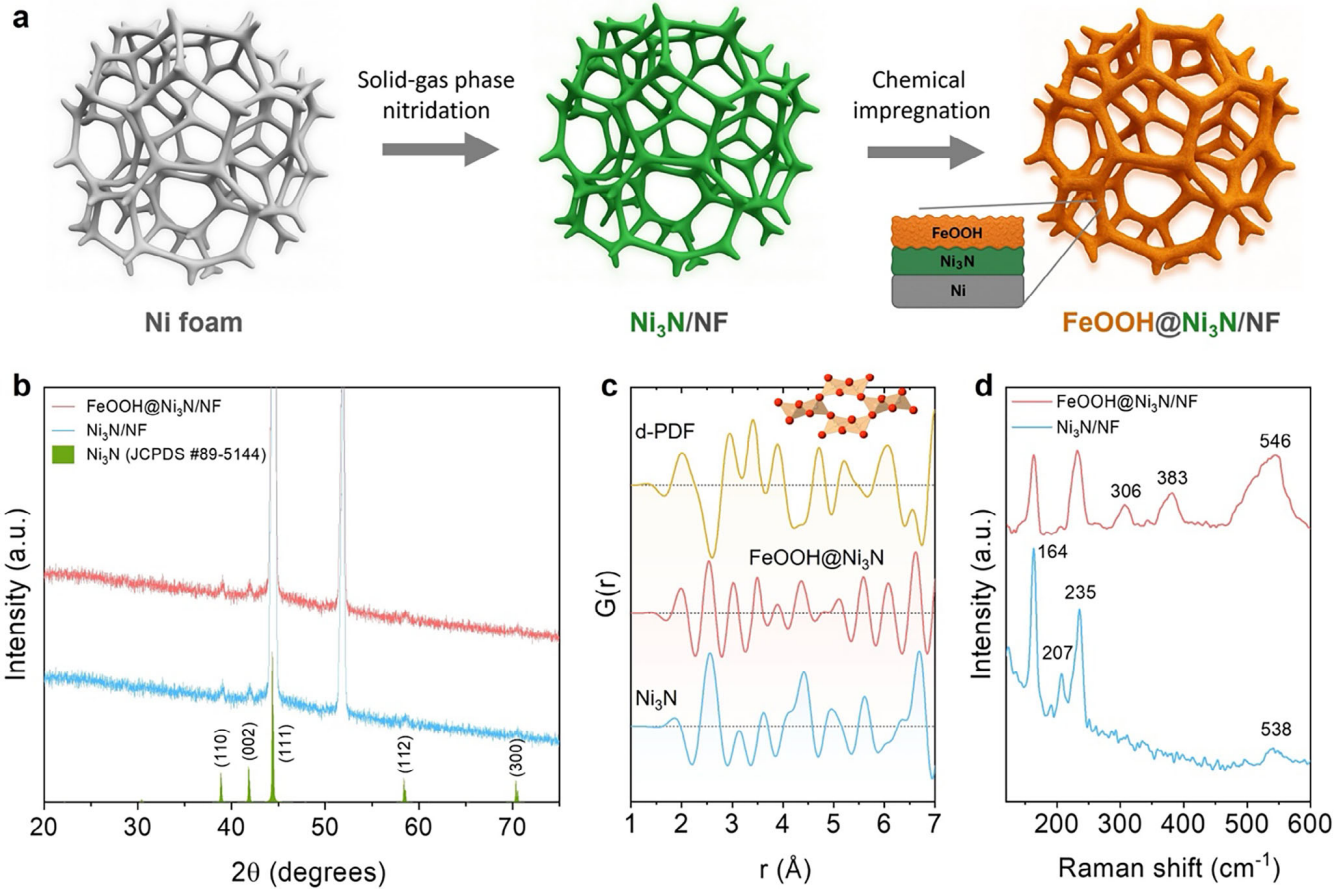
(a) Schematic illustration of the FeOOH@Ni_3_N/NF synthesis process. (b) XRD patterns of Ni_3_N/NF and FeOOH@Ni_3_N/NF. The strong diffraction peaks at 44.6° and 51.9° correspond to the (111) and (200) planes of the face‐centered cubic (*fcc*) Ni (JCPDS card no. 04‐0850). Standard diffraction data for hexagonal Ni_3_N (green lines, JCPDS card no. 89‐5144) are also given. (c) Reduced atomic pair distribution function G(r) of Ni_3_N, FeOOH@Ni_3_N/NF, and the differential PDF (d‐PDF) of FeOOH@Ni_3_N. (d) Raman spectra of the as‐prepared catalysts.

Figure 1a illustrates the stepwise synthesis of the FeOOH@Ni_3_N hybrid electrocatalyst via a combined solid‐gas nitridation and solution impregnation/oxidation route. First, a uniform Ni_3_N layer was directly grown on Ni(II)‐impregnated nickel foam (NF) by calcination under ammonia atmosphere, yielding Ni_3_N/NF. The in‐situ formation of the nitride phase on the 3D conductive scaffold minimizes interfacial resistance while ensuring intimate catalyst‐electrolyte contact and efficient mass transport. Subsequent short‐term immersion of Ni_3_N/NF in an aqueous iron(III) nitrate solution followed by mild heating at 80°C produced a conformal amorphous *α*‐FeOOH overlayer on the Ni_3_N backbone, yielding the FeOOH@Ni_3_N/NF heterostructure. The X‐ray diffraction (XRD) pattern of Ni_3_N/NF confirms the formation of crystalline hexagonal Ni_3_N (space group *P*6_3_22, JCPDS card no. 89‐5144), with characteristic reflections at 2*θ* ≈ 39.0° (110), 42.1° (002) and 58.5° (112) (Figure 1b). The well‐resolved peaks indicate good crystallinity and long‐range structural order, beneficial for efficient electron transport across the catalyst/support interface. After Fe deposition, the Ni_3_N reflections weaken due to the overlayer coverage, while no additional Fe‐related peaks are detected, implying that the Fe species are amorphous or ultra‐nanocrystalline, below the XRD detection limit. To probe the local atomic environments of the catalysts, high‐energy X‐ray total scattering coupled with pair distribution function (PDF) analysis was employed. This technique is sensitive to interatomic distance distribution and can provide detailed structural insight even for poorly crystalline or amorphous phases [37, 38, 39]. The PDF of Ni_3_N shows distinct interatomic correlations at 1.88 Å (Ni─N bonds), 2.56 Å (nearest‐neighbor Ni⋯Ni), and 3.14 and 3.62 Å (next‐nearest‐neighbor Ni⋯Ni/N) (Figure 1c), consistent with the hexagonal Ni_3_N structure (Figure S1). The short Ni⋯Ni distance (2.5–2.6 Å) reflects a close‐packed Ni sublattice, indicative of metallic‐like conductivity that is beneficial for electrocatalysis [40]. Differential PDF (d‐PDF) analysis, obtained by subtracting the Ni_3_N PDF profile from that of FeOOH@Ni_3_N (Figure 1c), reveals additional features assignable to orthorhombic *α*‐FeOOH (*Pnma*) (Figure S2) [41]. In particular, the correlations at 2.01 Å and 2.95 Å correspond to Fe─O bonds and Fe/O⋯Fe/O nearest‐neighbor distances, while peaks at 3.41 and 3.89 Å arise from next‐nearest‐neighbor Fe⋯Fe/O distances in the corner‐ and edge‐sharing FeO_3_(OH)_3_ octahedral network of *α*‐FeOOH. Raman spectroscopy further supports these assignments (Figure 1d). Ni_3_N/NF shows characteristic Ni–N lattice vibrations at 164, 207, 235 and 538 cm^−1^, [42] whereas FeOOH@Ni_3_N/NF exhibits additional *α*‐FeOOH bands at 306, 383 and 546 cm^−1^ [43]. Collectively, these results confirm the successful construction of a heterostructure comprising a crystalline, conductive Ni_3_N core intimately integrated with a conformal amorphous *α*‐FeOOH overlayer.

**FIGURE 2:**
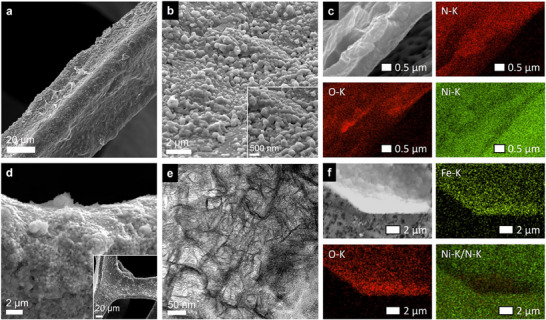
Representative SEM images of (a, b) Ni_3_N/NF and (d) FeOOH@Ni_3_N/NF at low and high magnification, illustrating the progressive surface morphology evolution during sequential synthesis. (e) TEM image of FeOOH@Ni_3_N/NF showing nano‐needle‐like *α*‐FeOOH domains. SEM‐EDS elemental mapping of (c) Ni_3_N/NF and (f) FeOOH@Ni_3_N/NF, indicating the underlying Ni_3_N core and the uniformly distributed *α*‐FeOOH shell.

The microstructure and morphology of the as‐synthesized electrodes were examined by field‐emission scanning electron microscopy (SEM) and transmission electron microscopy (TEM). SEM images reveal that Ni_3_N/NF consists of a uniform ≈400–450 nm‐thick Ni_3_N coating, conformally covering the Ni‐foam skeleton (Figure 2a; Figure S3). The Ni_3_N layer displays a nanogranular surface of densely packed nanoparticles, without visible cracks or delamination (Figure 2b), reflecting strong interfacial adhesion and efficient electron‐transport pathways throughout the electrode. Elemental mapping by SEM‐based energy‐dispersive X‐ray spectroscopy (EDS) confirms a Ni‐N‐rich composition with homogeneous distribution of Ni and N elements, along with minor oxygen signals, arising from superficial air oxidation into NiO_x_ species. (Figure 2c). Upon Fe incorporation, the electrode evolves into a hierarchical core–shell structure in which a compact *α*‐FeOOH layer (≈300 nm thick, Figure S4) conformally coats the conductive Ni_3_N backbone (Figure 2d). The *α*‐FeOOH overlayer comprises nanoscale grains with rough, porous morphology, providing plentiful surface‐active sites. Additional nanoscale insights from TEM imaging reveal that the *α*‐FeOOH domains adopt a needle‐like reticular morphology, with typical lengths of 50–70 nm and widths of 8–10 nm (Figure 2e) [44]. This high‐aspect‐ratio nanostructure imparts significant surface roughness and porosity, thereby facilitating electrolyte infiltration and promoting rapid O_2_ bubble release during OER operation. SEM‐EDS elemental mapping confirms the spatial distribution of Fe, Ni, N, and O elements, with Fe and O signals predominantly localized in the outer shell, while Ni and N are concentrated in the underlying core, unequivocally confirming the core‐shell architecture (Figure 2f). The intimate interfacial coupling between the conductive Ni_3_N core and the catalytically active *α*‐FeOOH shell ensures excellent electrical connectivity across the heterointerface, thereby enabling efficient charge transport during OER.

**FIGURE 3:**
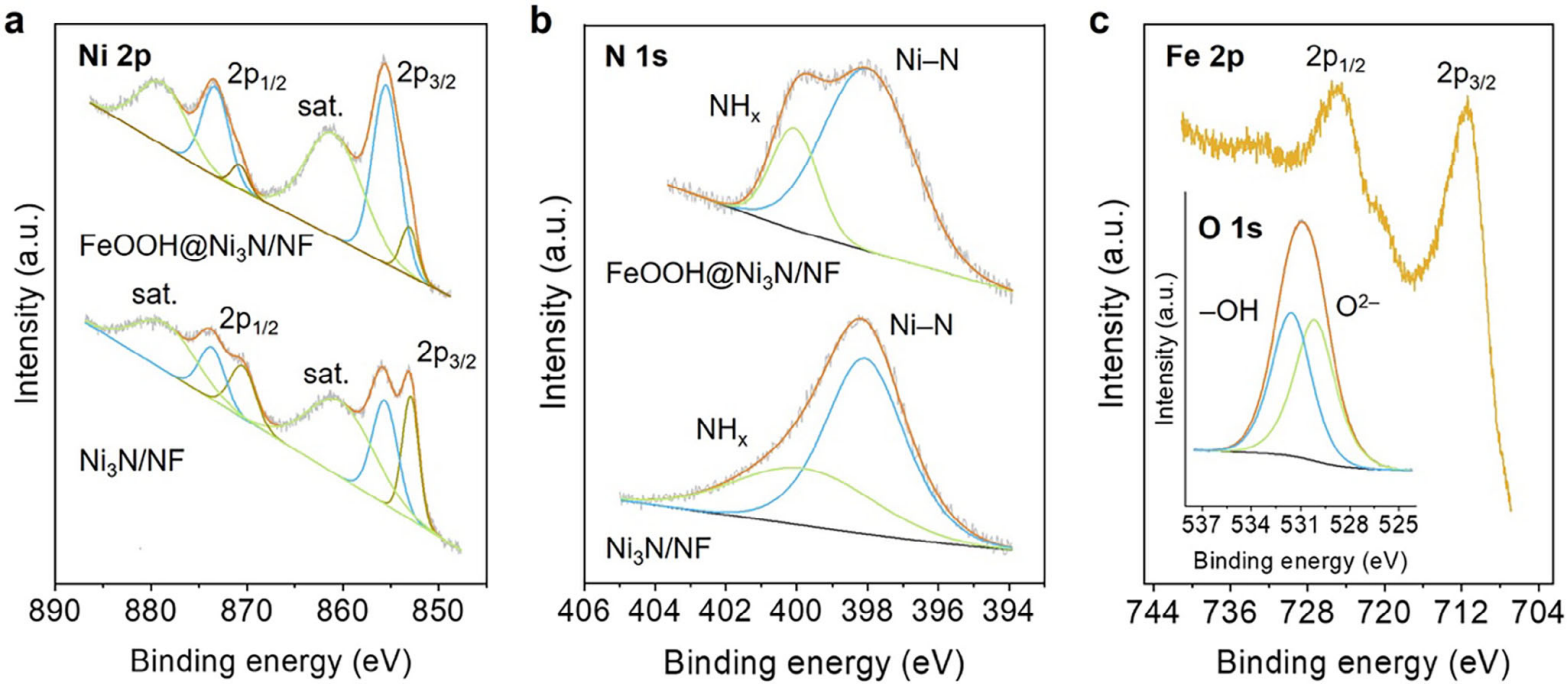
XPS spectra of (a) Ni 2*p* and (b) N 1*s* regions for Ni_3_N/NF (bottom traces) and FeOOH@Ni_3_N/NF (top traces). (c) XPS Fe 2*p* and O 1*s* (inset) spectra for FeOOH@Ni_3_N/NF. Deconvoluted peaks assigned to distinct chemical species are shown in blue, green, and yellow, while the orange curves represent the overall fitted envelopes.

X‐ray photoelectron spectroscopy (XPS) was employed to investigate the chemical state and surface element composition of the catalysts. For pristine Ni_3_N/NF, the Ni 2*p* spectrum displays two spin‐orbit doubles of Ni 2*p*_3/2_–2*p*_1/2_ at 852.9/870.4 eV and 855.6/873.6 eV, which can be attributed to the lattice Ni─N bonds and surface‐oxidized Ni–O species, respectively, indicating minor superficial oxidation of Ni_3_N upon air exposure (Figure 3a). The N 1*s* spectrum (Figure 3b) shows a dominant feature at 398.1 eV, corresponding to bulk Ni─N, along with a weaker peak at 399.0 eV associated with surface N–H/NH_x_ moieties formed during incomplete ammonolysis in the nitridation step [45]. After Fe deposition, the Ni 2*p* spectrum retains the Ni–N doublet at 852.9/870.5 eV (Figure 3a), confirming preservation of the Ni_3_N core. In contrast, the Ni–O doublet exhibits a negative binding‐energy shift of ≈0.3–0.4 eV (to 855.3/873.2 eV), reflecting strong electronic coupling between *α*‐FeOOH and Ni_3_N. This shift is consistent with interfacial electron transfer from *α*‐FeOOH to Ni_3_N, likely mediated by Fe–O–Ni linkages. The associated electron depletion in *α*‐FeOOH is expected to downshift its Fermi level and enhance its electrophilic character, features that favor OER‐related charge transfer processes. The N 1*s* spectrum of FeOOH@Ni_3_N/NF remains nearly unchanged, with peaks at 398.0 eV (metal‐N bonds) and 400.0 eV (surface NH_x_ species), further confirming core integrity (Figure 3b). Meanwhile, the Fe 2*p* spectrum shows a distinct 2*p*_3/2_–2*p*_1/2_ spin‐orbit doublet at 711.5 and 725.0 eV (Figure 3c), characteristic of Fe^3+^ in *α*‐FeOOH. The O 1*s* spectrum (Figure 3c, inset) can be deconvoluted into two peaks at 530.2 eV (lattice Fe–O) and 531.6 eV (surface Fe–OH), with a Fe–O/Fe–OH ratio of ≈1.04, very close to the expected 1:1 stoichiometry of *α*‐FeOOH. Quantitative XPS analysis of the Ni 2*p* and Fe 2*p* regions yields a Ni:Fe atomic ratio of ≈0.41:0.59, corresponding to ≈78 wt.% FeOOH within the XPS probing depth. These results confirm that the outer surface of FeOOH@Ni_3_N/NF is dominated by *α*‐FeOOH, while the conductive Ni_3_N core remains embedded beneath the overlayer, forming a robust core‐shell heterostructure.

### 2.2 Electrocatalytic Performance Testing

**FIGURE 4:**
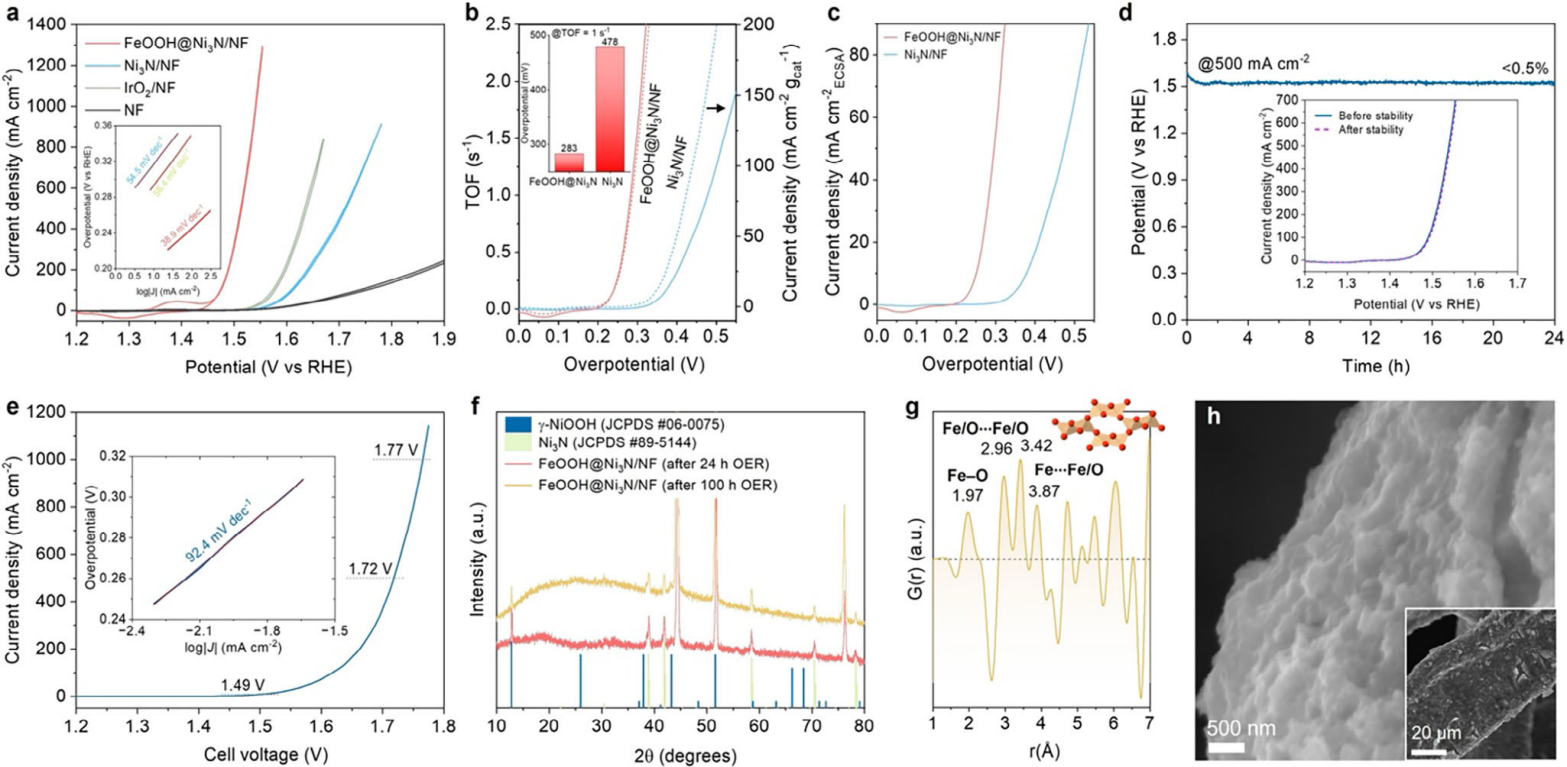
(a) *iR*‐corrected OER polarization curves of FeOOH@Ni_3_N/NF, Ni_3_N/NF, IrO_2_/NF, and bare Ni foam in 1.0 M KOH. Inset: corresponding Tafel plots (red lines: linear fits). (b) Potential‐dependent TOF and mass activity, and (c) ECSA‐normalized current density plots of FeOOH@Ni_3_N/NF and Ni_3_N/NF. (d) Chronopotentiometric stability of FeOOH@Ni_3_N/NF at 500 mA cm^−2^. Inset: OER polarization curves before and after the stability test. (e) Overall water‐splitting performance of a two‐electrode cell using FeOOH@Ni_3_N/NF as the anode and Ni foam as the cathode. Inset: corresponding Tafel plot at low potentials. (f) XRD patterns of the post‐OER catalysts collected after 24 and 100 h stability tests. (g) Differential PDF (d‐PDF) obtained by subtracting the Ni_3_N/NF PDF from that of post‐OER FeOOH@Ni_3_N/NF after 24 h of stability testing. (h) Representative SEM images of FeOOH@Ni_3_N/NF after 24 h of continuous electrolysis at 500 mA cm^−2^.

The electrocatalytic OER activity of FeOOH@Ni_3_N/NF was evaluated in O_2_‐saturated 1.0 M KOH using cyclic voltammetry (CV) by a standard three‐electrode setup. For comparison, measurements were also performed on pristine Ni_3_N/NF, commercial IrO_2_ catalyst supported on Ni foam (IrO_2_/NF), and bare Ni foam (NF). All potentials were calibrated against the reversible hydrogen electrode (RHE) and corrected for *iR* drop. To avoid interference from overlapping redox signals of metal species, the OER activity was quantified from the backward scan of the CV curves. As shown in Figure 4a, FeOOH@Ni_3_N/NF exhibits outstanding OER activity, requiring overpotentials of only 209 and 245 mV to deliver current densities of 10 and 100 mA cm^−2^, respectively – dramatically lower than Ni_3_N/NF (316 and 382 mV) and even surpassing IrO_2_ (291 and 347 mV). Strikingly, FeOOH@Ni_3_N/NF sustains industrial‐level operation, reaching 500 and 1000 mA cm^−2^ at overpotentials of 284 and 311 mV, respectively, demonstrating excellent high‐current capability for practical alkaline electrolysis. In contrast, bare NF shows negligible OER activity, confirming that both the *α*‐FeOOH shell and Ni_3_N core contribute to the catalytic response. Kinetic analysis at low overpotentials (Figure 4a, inset) reveals a markedly lower Tafel slope of 38.9 mV dec^−1^ for FeOOH@Ni_3_N/NF compared to 54.5 mV dec^−1^ for Ni_3_N/NF and 56.4 mV dec^−1^ for IrO_2_. This reflects a distinct potential‐determining step and more rapid reaction kinetics at the Fe–Ni heterointerface. The performance of FeOOH@Ni_3_N/NF compares favorably with, and often exceeds, best‐in‐class OER catalysts, including noble‐metal‐based and Ni‐Fe‐Co‐based materials (Table S1). Systematic optimization revealed that the best‐performing FeOOH@Ni_3_N/NF catalyst was obtained by immersing Ni foam in 15 mM Ni(NO_3_)_2_ solution for 2 min, followed by nitridation at 500°C, and subsequent impregnation in 15 mM Fe(NO_3_)_3_ solution for 10 min (Figures S5–S7). To assess intrinsic catalytic activity, the turnover frequency (TOF) was estimated based on the number of electrochemically active sites. The quantity of redox‐active sites for FeOOH@Ni_3_N/NF (1.30 × 10^−6^ mol cm^−2^) and Ni_3_N/NF (1.23 × 10^−6^ mol cm^−2^), determined through an electrochemical method, were found to be comparable, indicating similar active‐site densities (Figure S8) [46]. Nevertheless, site‐normalized TOF values (Figure 4b) reveal that FeOOH@Ni_3_N/NF exhibits significantly higher intrinsic activity than Ni_3_N/NF across the entire overpotential range, reaching 1.0 s^−1^ at an overpotential of 283 mV, whereas Ni_3_N/NF requires 478 mV to achieve the same TOF. This demonstrates that activity enhancement stems from interfacial electronic effects rather than increased surface site density. Mass activity analysis further confirms the efficiency advantage of FeOOH@Ni_3_N/NF, which delivers 150 A g^−1^ at an overpotential of 312 mV, representing a 161 mV improvement over Ni_3_N/NF (473 mV) at the same activity (Figure 4b). This substantial enhancement underscores the pivotal role of the Fe–Ni interfacial coupling in accelerating OER kinetics. The electrochemically active surface area (ECSA) was estimated from the double‐layer capacitance (C_dl_) determined by CV in the non‐Faradaic region (Figure S9). Through this analysis, the calculated ECSA values were 14.4 cm^2^ for FeOOH@Ni_3_N/NF and 9.1 cm^2^ for Ni_3_N/NF, suggesting only a modest increase in accessible electrocatalytic surface area upon Fe incorporation. After normalizing the current densities to ECSA, FeOOH@Ni_3_N/NF still retains a pronounced intrinsic advantage (Figure 4c), delivering 1 mA cm_ECSA_^−2^ at 213 mV compared with 314 mV for Ni_3_N/NF. Collectively, these results confirm that the performance boost of FeOOH@Ni_3_N/NF arises not from an increased number of surface‐active sites, but from synergistic electronic interactions between the conductive Ni_3_N core and the redox‐active *α*‐FeOOH shell, which jointly facilitate charge transfer and optimize intermediate adsorption energetics.

FeOOH@Ni_3_N/NF also demonstrates exceptional electrochemical durability under alkaline OER conditions. In a multistep chronopotentiometry test, where the current density was sequentially increased from 50 to 400 mA cm^−2^ in 50 mA cm^−2^ increments (1 h per step), the catalyst showed negligible performance loss (Figure S10), evidencing robust operation under fluctuating current loads. Furthermore, the potential rapidly stabilized after each current step, indicating efficient transport of OH^−^ ions and prompt release of evolved O_2_ bubbles from the catalyst surface, enabled by the open 3D mesh architecture of the electrode. At a constant industrially relevant current density of 500 mA cm^−2^, FeOOH@Ni_3_N/NF sustained stable operation for 24 h with less than 0.5% voltage increase (Figure 4d), while its post‐test CV curve nearly overlapped with the initial one (Figure 4d, inset), further attesting to its excellent structural and electrochemical integrity under high‐current conditions. In addition, during continuous operation at 10 mA cm^−2^, the catalyst maintained its activity for 72 h with only a 0.3% overpotential increase (Figure S11). Even after an extended 100 h durability test at a geometric current density of 500 mA cm^−2^, the catalyst retained remarkable stability, exhibiting a potential rise of only 1.2% (Figure S12). The OER polarization curves recorded before and after durability testing were nearly identical (Figure S12, inset). Collectively, these results demonstrate the exceptional durability and operational reliability of FeOOH@Ni_3_N/NF, underscoring its promise for practical, large‐scale alkaline water electrolysis.

Considering its outstanding OER activity, FeOOH@Ni_3_N/NF was further evaluated as the anode in a two‐electrode alkaline water electrolyzer using Ni foam as the cathode. The steady‐state polarization curve of the FeOOH@Ni_3_N/NF(+)||Ni foam(–) cell is presented in Figure 4e. Remarkably, this electrolyzer requires a low cell voltage of 1.49 V to deliver a water‐splitting current density of 10 mA cm^−2^, corresponding to an energy conversion efficiency of 82.6%. The low overall Tafel slope (92.4 mV dec^−1^) indicates favorable reaction kinetics on both electrodes (Figure 4e, inset). Notably, this performance surpasses noble‐metal‐based benchmark systems, including IrO_2_(+)||Pt/C(–) (1.58 V) [47], IrO_2_(+)||Pt(–)(1.57 V)[48] and RuO_2_(+)||Pt/C(–) (1.54 V) [49], as well as most recently reported high‐performance non‐noble‐metal catalysts that typically require >1.5 V under comparable conditions (Table S2). Strikingly, the FeOOH@Ni_3_N/NF(+)||Ni foam(–) electrolyzer sustains industrial‐level current densities of 500 and 1000 mA cm^−2^ at only 1.72 and 1.78 V, respectively, in 1.0 M KOH at 25°C. These voltages are significantly lower than those of conventional industrial electrodes, such as the standard Ni foam(–)||stainless steel(+) configuration, which typically demands 1.8–2.4 V to reach 200–500 mA cm^−2^ [32, 50], and even outperform advanced multi‐metallic catalysts that usually require ≥1.75 V to deliver 500 mA cm^−2^ (Table S2). Together, these results establish FeOOH@Ni_3_N/NF as a cost‐effective, durable, and high‐rate electrocatalyst capable of driving alkaline water electrolysis at industrially relevant current densities with superior energy efficiency.

To evaluate the structural and chemical stability of FeOOH@Ni_3_N/NF after prolonged OER operation, comprehensive post‐catalysis characterizations were conducted. For bulk structural analysis, the catalyst was carefully detached from Ni foam for total X‐ray scattering, while intact electrodes were examined by XRD, SEM and XPS. XRD patterns reveal partial surface reconstruction of the Ni_3_N core, with distinct *γ*‐NiOOH reflections emerging alongside residual Ni_3_N peaks (Figure 4f). This observation is consistent with previous reports that Ni_3_N undergoes surface oxidation under alkaline OER conditions, forming Ni(OH)_2_/NiOOH species containing catalytically active Ni^3^⁺^δ^–oxo sites [51, 52, 53, 54]. PDF analysis further confirms that the *α*‐FeOOH framework remains structurally intact after the 24 h OER durability test at 500 mA cm^−2^ (Figure 4g), as evidenced by the preservation of characteristic interatomic distances at 1.97 Å (Fe─O bonds), 2.96 Å (Fe/O⋯Fe/O nearest neighbors), and 3.42 and 3.87 Å (Fe⋯Fe/O next‐nearest neighbors). A slight contraction of the Fe─O bond (≈0.04 Å) is observed, likely associated with reversible Fe^3+^/Fe^4+^ redox cycling or interfacial Fe–O–Ni linkage formation during catalysis, without evidence of phase decomposition. SEM imaging corroborates the structural integrity of the electrode (Figure 4h and Figure S13). The *α*‐FeOOH overlayer remains continuous and firmly adhered to the Ni_3_N‐modified Ni foam, exhibiting only minor stress‐relief microcracks attributed to redox‐induced volume fluctuations. Higher magnification images reveal a wrinkled, cauliflower‐like morphology for *α*‐FeOOH, which enhances surface roughness and electrolyte accessibility while maintaining electronic connectivity with the underlying Ni_3_N core. Cross‐sectional SEM analysis shows a reconstructed shell of ≈300 nm thickness (Figure S13), consistent with the original architecture. SEM‐EDS mapping confirms Fe and O enrichment in the reconstructed outer layer, while Ni remains homogeneously distributed throughout the inner backbone (Figure S14). Interestingly, residual K⁺ cations are uniformly detected across the Fe oxyhydroxide shell, suggesting its direct involvement in the OER process. Post‐OER XPS analysis provides additional insight into surface chemistry evolution. The Fe 2*p* spectrum shows a Fe 2*p*_3/2_–2*p*_1/2_ doublet at 712.0 and 725.3 eV (Figure S15a), characteristic of Fe^3+^ in *α*‐FeOOH. Moreover, the O 1s region displays two deconvoluted peaks at 530.6 and 531.5 eV (Figure S15a, inset), corresponding to Fe–O lattice oxygen and Fe–OH species, respectively. The Ni 2*p* spectrum exhibits a positive binding‐energy shift (Ni 2*p*_3/2_: 855.6 eV, Ni 2*p*_1/2_: 873.3 eV) relative to the pre‐activated catalyst (Figure S15b), reflecting partial oxidation of interfacial Ni sites, in agreement with XRD evidence of *γ*‐NiOOH formation. In the N 1*s* region (Figure S15c), the upshifted peak at 399.1 eV indicates higher‐valence lattice N, likely arising from diminished Ni‐to‐N back‐donation upon surface Ni oxyhydroxide reconstruction and the emergence of O─Ni─N interfacial bonding. Near‐surface quantitative analysis of the Fe 2*p* and Ni 2*p* regions reveals a nearly unchanged Fe:Ni ratio after OER (59.0 at.% Fe vs. 59.4 at.% Fe before OER), within the XPS uncertainty, confirming excellent compositional stability. Collectively, these results indicate that while the Ni_3_N core undergoes partial surface oxidation to *γ*‐NiOOH, the *α*‐FeOOH shell remains chemically intact and firmly anchored, preserving a robust core‐shell architecture. This stable heterointerface reconstruction ensures long‐term durability, facilitates efficient charge transfer, and sustains high OER activity under industrially relevant current densities.

### 2.3 Active‐Site Evolution and Mechanistic Study

**FIGURE 5:**
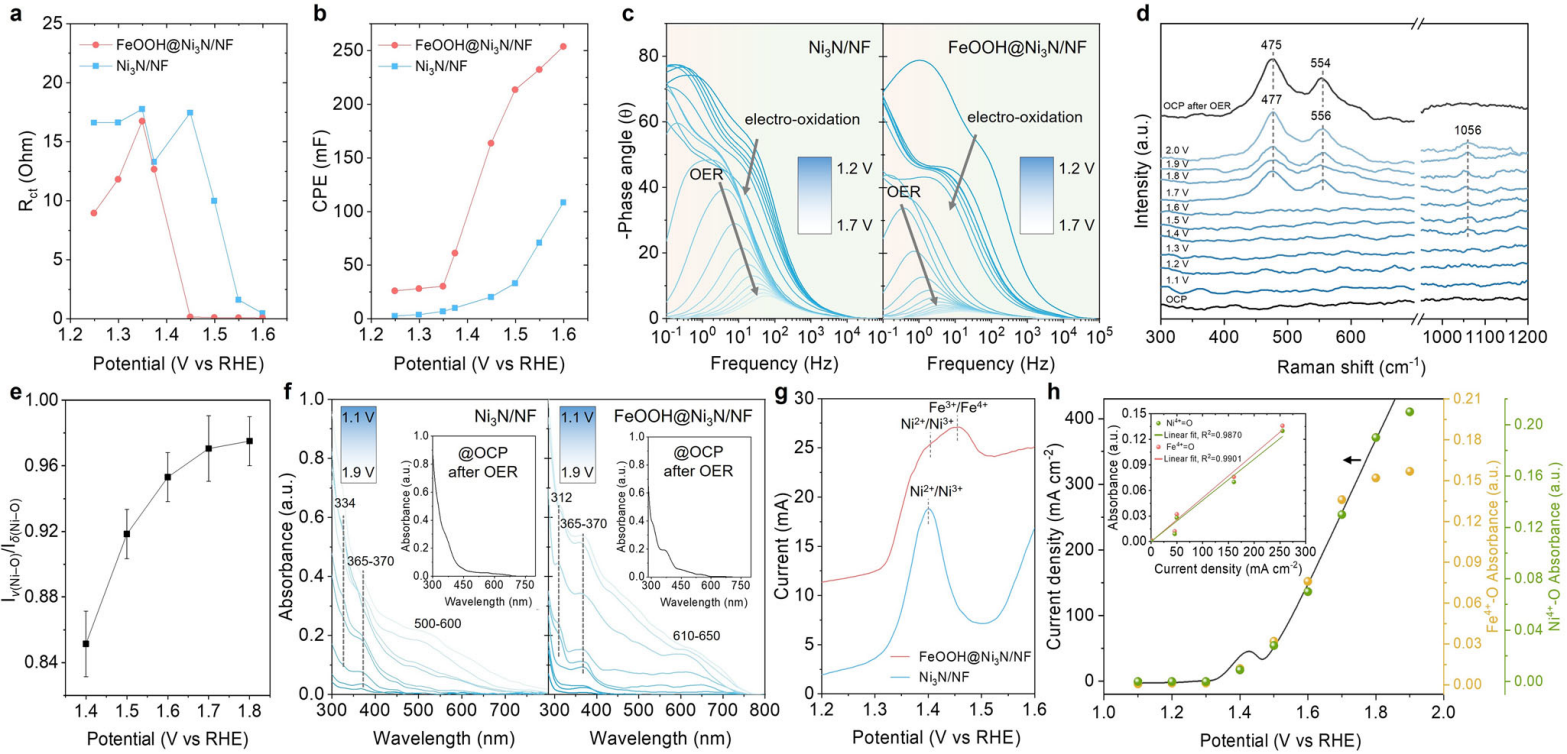
Potential‐dependent (a) interfacial charge‐transfer resistance (*R_ct_*) and (b) pseudocapacitive adsorption parameter (*CPE_ad_*) of FeOOH@Ni_3_N/NF and Ni_3_N/NF derived from operando EIS. (c) Operando Bode phase diagrams recorded at various potentials in O_2_‐saturated 1.0 M KOH for FeOOH@Ni_3_N/NF and Ni_3_N/NF. (d) In‐situ Raman spectra of FeOOH@Ni_3_N/NF collected from open‐circuit potential (OPC) to 2.0 V vs RHE. (e) Evolution of the I*_ν_*_(Ni–O)_/I*_δ_*_(Ni–O)_ intensity ratio of FeOOH@Ni_3_N/NF as a function of potential. (f) In situ UV–vis spectra of Ni_3_N/NF and FeOOH@Ni_3_N/NF under increasing potentials (insets: the UV–vis spectra at OCP after OER). (g) Differential pulse voltammetry (DPV) of FeOOH@Ni_3_N/NF and Ni_3_N/NF (scan rate: 50 mV s^−1^, 1.0 M KOH). (h) Correlation between current density (non‐*iR*‐corrected) and normalized absorbance at 550 nm (Ni^4+^ = O signature) and 610–650 nm (Fe^4+^ = O signature), highlighting the potential‐dependent generation of active species. Inset: absorbance‐current plots showing a linear correlation between the population of high‐valent Ni^4+^/Fe^4+^ species and OER current density.

To elucidate the dynamic electrochemical processes governing OER at the electrode/electrolyte interface, operando electrochemical impedance spectroscopy (EIS) was conducted over the potential range of 1.20–1.70 V vs. RHE in O_2_‐saturated 1.0 M KOH. The impedance spectra were collected in the frequency range of 100 kHz to 0.1 Hz, enabling real‐time monitoring of both charge‐transfer and adsorption‐related phenomena. Nyquist plots reveal a more pronounced and systematic decrease in the high‐frequency semicircle for FeOOH@Ni_3_N/NF relative to pristine Ni_3_N/NF (Figure S16a,b), signifying enhanced charge‐transfer efficiency and faster intermediate adsorption kinetics upon Fe incorporation. The EIS data were fitted using an adsorption‐enabled Armstrong‐Henderson equivalent circuit (Figure S16c), in which *R_ct_* represents charge‐transfer resistance and *CPE_ad_* corresponds to the pseudocapacitive response associated with adsorbed oxygenated intermediates. The fitted parameters are summarized in Table S3. As shown in Figure 5a, FeOOH@Ni_3_N/NF exhibits a markedly earlier and steeper decline in *R_ct_*, initiating at 1.35 V vs. RHE, whereas Ni_3_N/NF shows a comparable drop only above 1.45 V vs. RHE. Moreover, throughout the 1.25–1.60 V vs. RHE range, FeOOH@Ni_3_N/NF consistently maintains lower *R_ct_* values, confirming superior cross‐interfacial charge transport and earlier OER activation. A similar trend is observed in the evolution of *CPE_ad_* (Figure 5b). Across the entire potential range, FeOOH@Ni_3_N/NF displays higher *CPE_ad_* values than Ni_3_N/NF, reflecting more efficient adsorption and turnover of oxygenated intermediates at the Fe–Ni heterointerface. These observations collectively indicate that Fe incorporation fundamentally modulates the electrode's surface electronic structure, facilitating faster intermediate binding and more efficient interfacial charge exchange. Bode phase plots further substantiate these dynamic effects (Figure 5c). For pristine Ni_3_N/NF, a prominent high‐frequency (>10 Hz) phase maximum gradually diminishes between 1.20–1.40 V vs. RHE, indicative of sluggish surface electrooxidation and limited *OH coverage. In contrast, FeOOH@Ni_3_N/NF exhibits a more rapid suppression of this feature within the same potential window, signifying accelerated OH^−^ adsorption and greater *OH accumulation, which favor OER initiation. Concurrently, between 1.20–1.40 V vs. RHE, FeOOH@Ni_3_N/NF displays a moderated oxidation response relative to Ni_3_N/NF, suggesting that the α‐FeOOH overlayer effectively regulates Ni‐site oxidation and promotes interfacial reaction kinetics. At higher potentials, both catalysts show a frequency shift of the dominant phase‐angle response to below 10 Hz, marking the transition from surface oxidation to charge‐transfer‐limited OER regime. Notably, this transition occurs earlier for FeOOH@Ni_3_N/NF (1.42 V) than for Ni_3_N/NF (>1.50 V), further highlighting premature catalytic activation induced by Fe incorporation. Moreover, FeOOH@Ni_3_N/NF exhibits a larger low‐frequency phase‐angle decrease, consistent with faster OER kinetics and more efficient charge dissipation through the FeOOH/Ni_3_N interface [55]. Collectively, operando EIS and Bode analyses reveal a cooperative electronic and catalytic synergy at the Fe–Ni heterointerface. The *α*‐FeOOH shell provides abundant redox‐active sites that optimize adsorption energetics and turnover of oxygenated intermediates (manifested by higher *CPE_ad_*), while simultaneously lowering the interfacial charge‐transfer barrier (earlier *R_ct_* decrease). Meanwhile, the conductive Ni_3_N core ensures rapid electron transport, minimizing resistive losses during catalysis. The ensemble effect of this hierarchical, electronically coupled heterostructure thus facilitates a heterointerface‐accelerated OER pathway, accounting for the superior reaction kinetics and overall performance of FeOOH@Ni_3_N/NF compared to pristine Ni_3_N/NF.

To get in‐depth information on dynamic structure transformations during OER, in situ Raman and ultraviolet–visible (UV–vis) spectroscopy were performed as a function of applied potential. These complementary techniques allow direct observation of lattice reconstruction, intermediate formation, and redox evolution of Ni and Fe sites under operating conditions. Raman spectra were collected in a custom‐built electrochemical cell over the potential range 1.1–2.0 V vs. RHE. Upon anodic polarization to 1.3 V vs. RHE, the pristine Ni_3_N/NF displays two conspicuous vibrational bands at 484 and 562 cm^−1^, assigned to the *δ*(Ni–O) bending (E_g_) and *ν*(Ni–O) stretching (A_1g_) modes of nickel oxyhydroxide (Figure S17) [56, 57, 58]. According to literature, these features are attributed to *γ*‐NiOOH, which possesses weaker *ν*(Ni–O) intensity than *β*‐NiOOH due to its disordered and expanded lattice structure [59, 60, 61, 62, 63]. Notably, with increasing potential, these bands intensify but rapidly diminish after OER completion, indicating the transient formation and consumption of Ni^3+^/Ni^4+^ species. The reversible relaxation of surface‐localized Ni^4+^ = O back to Ni^3+^–oxo and its partial electrochemical dissolution into electrolyte reflects the metastable and corrosive nature of surface Ni oxyhydroxide, which can induce lattice strain and microcracking during prolonged cycling [64, 65, 66]. In contrast, FeOOH@Ni_3_N/NF exhibits *γ*‐NiOOH Raman bands at 477 and 556 cm^−1^ only above 1.7 V vs. RHE (Figure 5d), revealing that Fe incorporation delays Ni oxidation at the heterointerface. At higher potentials, these signals increase sharply, reflecting the rapid accumulation of Ni^3+^ species at high bias. The I*_ν_*_(Ni–O)_/I*_δ_*_(Ni–O)_ ratio, a measure of lattice disorder in nickel oxyhydroxide, [59] increases by ≈13% under OER bias for FeOOH@Ni_3_N/NF (Figure 5e), suggesting a more disordered oxyhydroxide structure caused by Fe‐induced oxygen vacancies and interfacial Fe^3+^–O–Ni^3+^ bonding [67, 68]. Importantly, after OER at open circuit conditions, FeOOH@Ni_3_N/NF retains distinct *γ*‐NiOOH features (475 and 554 cm^−1^), confirming that the *α*‐FeOOH overlayer stabilizes oxidized Ni^3+^ sites and increases the density of catalytically active species. Notably, no Raman peak at 525–535 cm^−1^ (Ni^2+^–O vibration in Ni(OH)_2_)[69] was detected for either catalyst, indicating rapid Ni^2+^ → Ni^3+^ oxidation under anodic bias. Above 1.4 V, FeOOH@Ni_3_N/NF also exhibits a broad Raman feature at 1050–1070 cm^−1^, attributable to surface‐bound hydroperoxo (*OOH) intermediates [70]. The potential‐dependent growth of this band, together with electrokinetic and electrolyte‐dependent studies (see below), supports an adsorbate evolution mechanism (AEM) involving the sequential formation of *OH, *O, and *OOH intermediates. Complementary in situ UV–vis spectroelectrochemistry was performed in a custom‐built cell over 1.1–1.9 V vs. RHE, with spectra referenced to the open‐circuit state. For Ni_3_N/NF, surface reconstruction initiates at 1.3 V vs. RHE, accompanied by absorptions at ≈334 nm and 365–370 nm, corresponding to O^2–^ → Ni^3+^ ligand‐to‐metal charge transfer (LMCT) transitions (Figure 5f). After OER, these features diminish but remain faintly detectable, signifying the presence of residual Ni^3+^ coloration centers that are too dilute for Raman detection. In the steady OER regime (>1.5 V), a broad 500–600 nm band emerges, characteristic of Ni^3+^/Ni^4+^ intervalence charge transfer (IVCT) and transient formation of Ni^4+^ = O [71, 72]. Its disappearance upon relaxation confirms Ni^4+^ = O as a short‐lived catalytic intermediate (Figure 5f, inset). FeOOH@Ni_3_N/NF displays similar LMCT features (≈312 nm and 365–370 nm) but with a delayed onset at 1.40 V (Figure 5f), consistent with suppression of premature Ni oxidation by Fe incorporation. Under OER conditions, the catalyst exhibits both Ni^3+^/Ni^4+^ IVCT (≈550 nm) and an additional broad band at 610–650 nm, attributed to Fe^3+^/Fe^4+^ IVCT within *α*‐FeOOH [73]. The simultaneous rise of these bands with increasing potential reveals cooperative redox activation of Ni and Fe centers at the heterointerfaces. To clearly depict surface structural changes, the Ni^3+^‐related absorbance (365–370 nm) was analyzed under varying potentials. While Ni_3_N/NF shows near‐linear oxidation with potential, FeOOH@Ni_3_N/NF exhibits relatively suppressed Ni oxidation at low bias and undergoes a sharp activation above 1.6 V (Figure S18), consistent with delayed *γ*‐NiOOH formation. Differential pulse voltammetry (DPV) further supports these observations (Figure 5g), showing two anodic features for FeOOH@Ni_3_N/NF: a shoulder at 1.35–1.40 vs. RHE, attributed to the Ni^2+^/Ni^3+^ transition, and a broader wave at ≈1.45 vs. RHE, arising from overlapping Fe^3+^/Fe^4+^ and Ni^3+^/Ni^4+^ redox couples [74, 75, 76]. The slight anodic shift (by 5–10 mV) of the Ni^2+^/Ni^3+^ peak upon Fe incorporation suggests electronic modulation of *γ*‐NiOOH, likely through the formation of Fe–O–Ni linkages [77]. Notably, pristine Ni_3_N/NF lacks a distinct Ni^3+^/Ni^4+^ feature, confirming that the heterointerface is beneficial to stabilize high‐valent Ni^4+^ species through Fe–O–Ni‐driven charge delocalization [77, 78]. Importantly, the transient Ni^4+^ = O (≈550 nm) and Fe^4+^ = O (≈610–650 nm) spectra signatures scale linearly with OER current density (Figure 5h), demonstrating that the buildup of high‐valent metal‐oxo species is kinetically coupled to catalytic turnover. Together with in‐situ Raman data, these results confirm that OER on FeOOH@Ni_3_N/NF proceeds via a heterointerface‐driven AEM pathway. The *α*‐FeOOH overlayer stabilizes oxidized Ni^3+^/Ni^4+^ sites, extends their lifetime, and introduces electrophilic Fe^3+^/Fe^4+^ centers that cooperate with Ni^4+^ = O to promote HO^–^ adsorption and O─O bond formation. This dual‐site mechanism underlies the superior OER activity and stability of FeOOH@Ni_3_N/NF compared to pristine Ni_3_N/NF.

**FIGURE 6:**
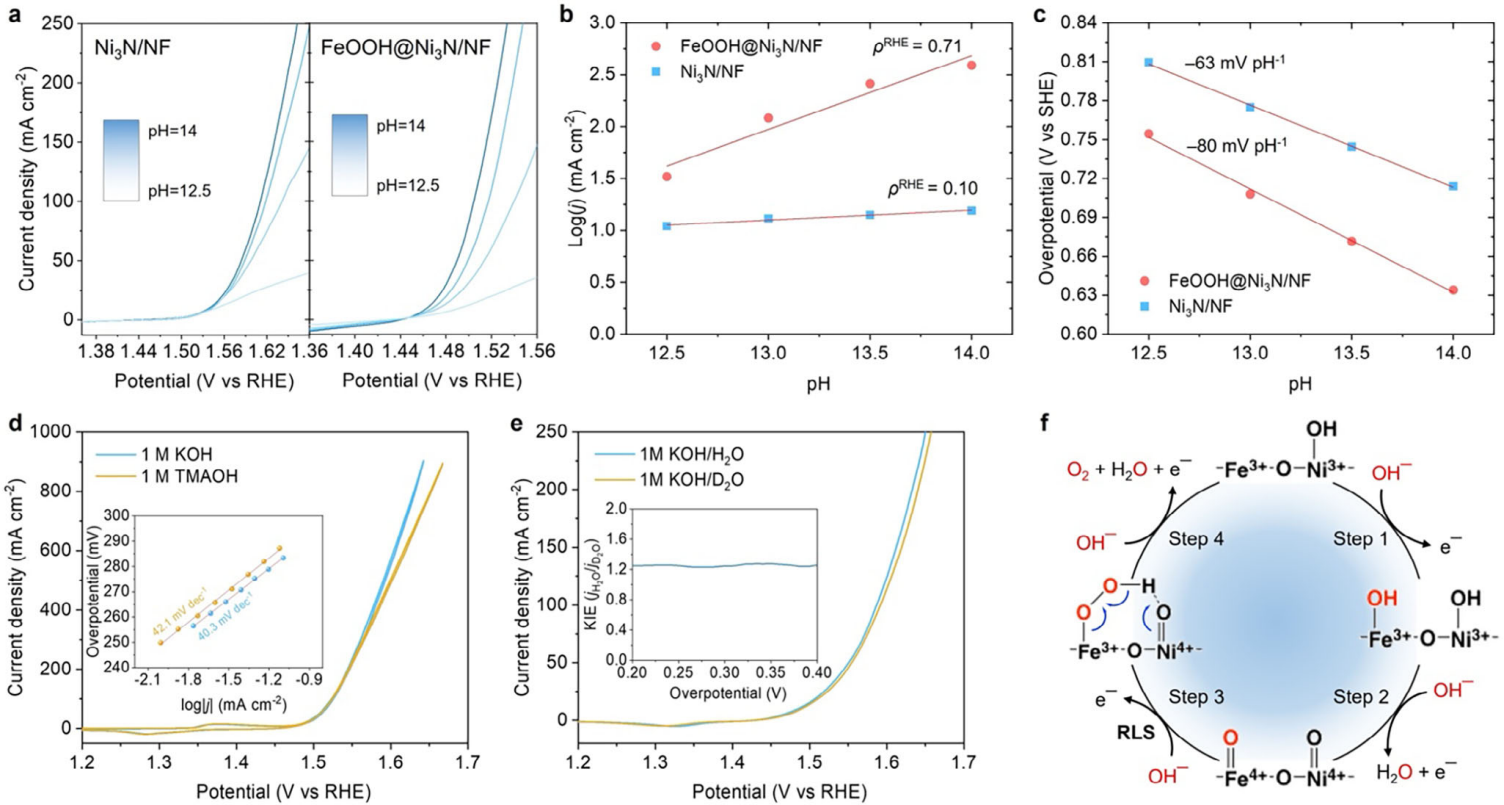
(a) OER activity plots (*iR*‐corrected), (b) logarithms of current density at 1.55 V vs RHE, and (c) overpotentials at 10 mA cm^−2^ (SHE scale) as a function of electrolyte pH for FeOOH@Ni_3_N/NF and Ni_3_N/NF. (d) CV curves and corresponding Tafel plots (inset) of FeOOH@Ni_3_N/NF in 1.0 M KOH and 1.0 M TMAOH, illustrating negligible cation effects. (e) Polarization curves of FeOOH@Ni_3_N/NF in 1.0 M KOH/H_2_O and 1.0 M KOH/D_2_O solution and the corresponding kinetic isotope effect (KIE) plot (inset), confirming secondary isotope effect. (f) Proposed dual‐site OER mechanism at the Fe–Ni heterointerface involving a decoupled PET pathway over the Fe^4+^ = O and Ni^4+^ = O active sites, leading to enhanced OER kinetics and stability of the FeOOH@Ni_3_N/NF catalyst.

To elucidate the proton‐electron transfer (PET) kinetics governing the OER, systematic pH‐dependent electrocatalytic studies were conducted in the pH range of 12.5–14 (Figure 6a). The Ni_3_N/NF catalyst exhibits nearly invariant OER activity with increasing OH^−^ concentration, yielding a low proton reaction order (*ρ* ^RHE^ ≈ 0.10, Figure 6b) and a near‐Nernstian slope of –63 mV pH^−1^ on the SHE scale (Figure 6c). Such behavior is consistent with a concerted PET pathway, in which *OH deprotonation or *O oxidation on *γ*‐NiOOH constitutes the rate‐limiting step (RLS) [56]. In stark contrast, FeOOH@Ni_3_N/NF displays pronounced pH‐dependent OER activity, with *ρ* ^RHE^ ≈ 0.71 and a super‐Nernstian slope of –80 mV pH^−1^. These features indicate a 3OH^−^/2e^−^ exchange process governed by a decoupled PET mechanism, wherein nucleophilic OH^–^ attack drives O─O bond formation as the RLS [56, 79, 80]. To further discriminate among possible mechanisms, the OER activity of FeOOH@Ni_3_N/NF was compared in 1.0 M KOH and 1.0 M tetramethylammonium hydroxide (TMAOH) electrolytes (Figure 6d). Bulky, weakly hydrated TMA^+^ cations strongly perturb the outer Helmholtz plane (OHP) hydrogen‐bond network; thus, if OHP solvation governed the RLS, marked activity variations would be expected [81, 82]. However, FeOOH@Ni_3_N/NF exhibits nearly identical performance in both electrolytes (Figure 6d), excluding OHP solvation effects and supporting an inner‐sphere hydroxyl nucleophilic attack (HNA) pathway [83]. Moreover, the negligible cation dependence rules out a lattice oxygen‐mediated mechanism (LOM), which typically manifests as enhanced activity upon cycling due to surface metal leaching and structural degradation [8, 84]. Consistent with this, FeOOH@Ni_3_N/NF demonstrates excellent durability, confirming robust surface reconstruction with minimal lattice oxygen exchange. To further probe possible proton‐transfer behavior, kinetic isotope effect (KIE) measurements were performed in 1.0 M KOH prepared with H_2_O and D_2_O. Given the slower deuterium transfer kinetics, a pronounced isotope effect would implicate direct O–H(D) bond cleavage as the RLS. Instead, FeOOH@Ni_3_N/NF shows only a modest isotope effect (*j*_H2O_/*j*_D2O_ ≈ 1.2–1.3, Figure 6e), excluding primary O─H bond cleavage as the RLS and confirming OH^−^ attack on electrophilic metal–oxo species as the critical step. Integrating these kinetic and spectroscopic results, FeOOH@Ni_3_N/NF follows a heterointerface‐driven AEM. The near‐first‐order OH^−^ dependence, super‐Nernstian slope, and modest KIE collectively point to a decoupled PET‐driven process, in which OH^−^ nucleophilically attacks Fe^4+^ = O to form a *OOH intermediate stabilized by adjacent Ni^4+^ = O sites (*O + OH^−^ → *OOH + e^−^). Consistent with this mechanism, in‐situ Raman spectroscopy captured the transient *OOH vibrational signature, while UV–vis spectroscopy revealed the synchronous evolution of Ni^4+^/Fe^4+^ intervalence charge‐transfer bands, directly correlating the accumulation of high‐valent metal‐oxo species with the OER current (Figure 5h). This dual‐site mechanism is further reinforced by strong electronic coupling across Fe–O–Ni linkages between *α*‐FeOOH and conductive Ni_3_N, which facilitates charge delocalization and lowers the activation barrier for O─O bond formation. Additional evidence arises from Tafel slope analysis as a function of electrolyte pH, where FeOOH@Ni_3_N/NF shows a distinct shift from 36.2 to 32.0 mV dec^−1^ with increasing pH, in contrast to Ni_3_N/NF, which remains essentially invariant (60.3–61.6 mV dec^−1^) (Figure S19). This pH‐sensitive behavior reflects altered *OH/*O energetics at the heterointerface, consistent with the HNA mechanism [85, 86]. Based on these findings, the mechanistic steps over FeOOH@Ni_3_N/NF are summarized in Figure 6f. Under anodic bias (>1.3 vs RHE), Ni_3_N partially oxidizes to *γ*‐NiOOH. During OER, OH^−^ adsorption and deprotonation at Ni^3+^/Fe^3+^ sites yield highly electrophilic Ni^4+^ = O and Fe^4+^ = O Lewis acid centers as pre‐equilibrium intermediates. Nucleophilic OH^−^ then attacks Fe^4+^ = O, producing a Fe^3+^–OOH intermediate that bridges to adjacent Ni^3+^–O/Ni^4+^ = O centers to form a *µ*‐hydroperoxo (Fe^3+^–OOH⋅⋅⋅O–Ni^3+/4+^) species stabilized by intermolecular hydrogen bonding; this constitutes the RLS. Here, the partial O─H bond cleavage occurring across two adjacent metal‐oxo sites is consistent with the modest KIE observation. Subsequently, through decoupled PET steps, *OOH deprotonates and oxidizes, releasing O_2_ and regenerating Fe^3+^/Ni^3+^ active sites. In this dual‐site cooperative mechanism, Fe^4+^ = O acts as the electrophilic center, accepting the lone pair of OH^−^, whereas Ni^4+^ = O, being relatively less electron‐deficient, functions as a proximal redox partner that stabilizes the *µ*‐hydroperoxo transition state through charge delocalization. This dynamically coupled dual‐site OER pathway effectively circumvents the linear adsorption‐energy scaling relationship among *OH, *O, and *OOH intermediates inherent to single‐site catalysts, which limit their intrinsic activity [87, 88]. The synergistic division of roles between Fe and Ni lowers the activation barrier for O─O bond formation, thereby accelerating OER kinetics and imparting remarkable catalytic stability. This experimentally validated dual‐site mechanism is consistent with theoretical predictions for Fe–Ni‐based oxides and molecular complexes, which propose a low‐energy cooperative O─O coupling via nucleophilic OH^−^ attack on Fe^4+^ = O centers concurrent with hydrogen transfer to neighboring Ni–O moieties [89, 90, 91].

## 3 Conclusion

In summary, we have developed a hierarchical FeOOH@Ni_3_N/NF electrocatalyst that integrates a highly conductive Ni_3_N backbone with a redox‐active *α*‐FeOOH overlayer, achieving outstanding OER activity and durability under alkaline conditions. The catalyst requires overpotentials of only 209, 245, 284, and 311 mV to reach current densities of 10, 100, 500, and 1000 mA cm^−2^, respectively, and maintains exceptional stability at industrially relevant rates. Comprehensive operando/in situ Raman, UV–vis, and electrochemical spectroscopies, together with pH‐dependent kinetics, isotope probing, and cation‐effect studies, reveal that the superior performance arises from cooperative Fe–Ni interfacial chemistry. This synergy stabilizes high‐valent Ni^4+^ = O and Fe^4+^ = O species and promotes a decoupled proton‐electron transfer (PET)‐mediated adsorbate evolution mechanism (AEM), in which nucleophilic OH^−^ attack on Fe^4+^ = O generates the rate‐limiting *OOH intermediate, while neighboring Ni sites modulate the local charge density to facilitate O─O bond formation and O_2_ evolution. Benefiting from this unconventional dual‐site cooperated pathway, a two‐electrode alkaline water electrolyzer employing FeOOH@Ni_3_N/NF as the anode operates at a cell voltage of only 1.49 V to deliver 10 mA cm^−2^, and sustains commercially relevant current densities of 500 and 1000 mA cm^−2^ at 1.72 and 1.78 V, respectively, outperforming state‐of‐the‐art noble‐metal‐based benchmarks. This work establishes a clear structure–activity–stability relationship for FeOOH/Ni_3_N heterostructures and underscores heterointerface engineering as a powerful strategy for accelerating OER kinetics. More broadly, these findings offer a generalizable design blueprint for coupling conductive nitride scaffolds with catalytically active oxyhydroxide layers, paving the way toward cost‐effective, durable, and scalable alkaline water electrolyzers for sustainable hydrogen production.

## Author Contributions

M.M. carried out the synthesis of materials, structural characterizations, and electrocatalytic performance evaluation. I.V. performed EIS analysis and conducted the total X‐ray scattering and in situ Raman spectroscopy experiments. M.M., I.V., and G.S.A. contributed to the writing of the paper. G.S.A. conceived the concept, designed the study, and supervised the project.

## Conflicts of Interest

The authors declare no conflicts of interest.

## Supporting information




**Supporting File**: smll72007‐sup‐0001‐SuppMat.docx.

## Data Availability

The data that supports the finding of this study are available in the Supporting Information of this article or from the corresponding authors upon reasonable request.
