# Supplementary material for: Heterointerface Engineering of FeOOH@Ni3N Electrocatalysts for Industrially Compatible Alkaline Water Electrolysis
Source: Small. 2025 Dec 26;22(9):e13136. doi: 10.1002/smll.202513136 (PMC12895143; doi:10.1002/smll.202513136)
Supplement: Supplementary file 1 — Supporting File: smll72007‐sup‐0001‐SuppMat.docx. [file SMLL-22-e13136-s001.docx]

**Heterointerface Engineering of FeOOH@Ni_3_N Electrocatalysts for Industrially Compatible Alkaline Water Electrolysis**

Maria S. Metaxa, Ioannis Vamvasakis, Gerasimos S. Armatas*

Department of Materials Science and Engineering, University of Crete, Vassilika Vouton, Heraklion 70013, Greece

*Corresponding author. E-mail address: garmatas@uoc.gr

**Experimental Methods**

***Chemicals***

Iron(III) nitrate nonahydrate (Fe(NO_3_)_3_·(H_2_O)_9_, ≥98%), nickel(II) nitrate hexahydrate (Ni(NO_3_)_2_·6(H_2_O)_9_, ≥98%), hydrochloric acid (HCl, ≥37%, ACS reagent grade), iridium(IV) oxide powder (IrO_2_, 99.9% metal basis) and deuterium oxide (D_2_O, 99.8%) were purchased from Sigma-Aldrich. Tetraethylammonium hydroxide (10% in water, ~1.1 M) was obtained from Fluka. Ammonium hydroxide solution (~25% NH_3_ basis), ethanol (≥98%) and potassium hydroxide (KOH, ≥85%) were supplied by Honeywell. All chemical were used as received without further purification. Ni foam (1.5 mm thickness; 80-120 ppi) was purchased from Fuel Cell Store.

***Synthesis of the Ni_3_N/NF and FeOOH@Ni_3_N/NF electrocatalysts***

Nickel nitride (Ni_3_N) was growth on nickel foam (NF) substrates via a gas-phase nitridation process. Prior to synthesis, Ni foam pieces (1 × 2 cm^2^) were cleaned by sequential ultrasonication in 1.0 M HCl, deionized (DI) water, acetone and ethanol for 15 min each, followed by drying at 60 ^o^C for 12 h. The pretreated Ni foam was then immersed in an aqueous Ni(NO_3_)_2_ solution (15 mM, 10 mL) for 2 min at room temperature, gently rinsed with DI water to remove excess precursors, and dried at 60 °C for 1 h. The Ni-impregnated foam was then placed in an alumina boat and inserted into a tubular furnace. After purging with N_2_ for 10 min, the system was heated to 500 °C and maintained for 1 h under a continuous NH_3_ flow (~100 cm^3^ min^–1^), promoting the conversion of surface Ni species to Ni_3_N on the NF substrate. Under the examined conditions (400–550 °C, 1 h), the Ni foam retained its mechanical integrity and showed no signs of embrittlement. After natural cooling to room temperature under N_2_ atmosphere, the resulting Ni_3_N/NF electrodes were obtained. The Ni_3_N loading was determined to be ~3 mg cm^–2^.

In the following, the as-prepared Ni_3_N_2_/NF electrodes were modified with an α-FeOOH overlayer by a facile post-synthetic hydrolysis/condensation route. Specifically, Ni_3_N_2_/NF was immersed in an aqueous Fe(NO_3_)_3_ solution (10 mL, 15 mM) for 10 min at room temperature to enable surface adsorption of Fe^3+^ ions. Afterward, the electrode was gently rinsed with DI water to remove weakly bound species and dried at 80 ^o^C for 12 h under ambient conditions. This mild treatment induces in situ hydrolysis of surface-anchored Fe^3+^, yielding a conformal α-FeOOH shell on the Ni_3_N core. The total catalyst loading was found to be ~7 mg cm^–2^ for FeOOH@Ni_3_N/NF.

To tune catalyst loading and morphology, different impregnation times were tested for Ni(NO_3_)_2_ (1, 2 and 5 min) and Fe(NO_3_)_3_ (5, 10 and 15 min) precursors. The resulting electrodes were systematically evaluated to optimize synthetic parameters for maximal OER performance.

***Synthesis of the IrO_2_/NF electrocatalyst***

The IrO_2_/NF electrode was prepared by a drop-coating method. In a typical procedure, 7 mg of commercial IrO_2_ powder was dispersed in 500 μL ethanol and ultrasonicated for 60 min to form a homogeneous suspension. The resulting ink was then drop-cast onto a pre-cleaned nickel foam substrate (1 × 2 cm^2^) and dried in air at 60 ^o^C for 4 h to yield the IrO_2_/NF electrode.

***Electrocatalyst characterization***

X-ray diffraction (XRD) patterns were collected on a Rigaku SmartLab SE diffractometer using Cu Kα radiation (λ=1.5406 Å) operated at 40 kV and 50 mA. Total X-ray scattering measurements were performed at room temperature on a Bruker D8 Venture diffractometer equipped with a PHOTION II CPAD detector, using capillary transmission geometry and a Mo Kα source (λ=0.7093 Å). Data were corrected for empty cell scattering and the scattering intensity I(*q*) (where *q* = 4·π·sinθ/*λ*) was Fourier transformed using PDFgetX3 to obtain pair distribution function (PDF) plots.^[[1]](#endnote-1)^ Field-emission scanning electron microscopy (FE-SEM) and energy-dispersive X-ray spectroscopy (EDS) mapping were performed on a JEOL JSM-IT700HR microscope operated at 20 kV. Transmission electron microscopy (TEM) images were obtained on a JEOL JEM-2100 instrument with a LaB_6_ filament operated at 200 kV. TEM specimens were prepared by sonicating the catalyst powders in ethanol and drop-casting the suspension onto holey carbon-coated Cu grids. X-ray photoelectron spectroscopy (XPS) was conducted on a SPECs system equipped with a Phoibos 100 1D-DLD electron analyzer and Al Kα radiation (1486.6 eV). Binding energies were calibrated to the C 1s peak at 284.8 eV. In-situ UV-vis absorption spectra were recorded on a Shimadzu UV-2600 spectrometer (250–800 nm) using a custom-built spectroelectrochemical cell (1 cm optical path) equipped with the sample-coated working electrode, a Hg/HgO reference electrode (1.0 M KOH), and a Pt wire counter electrode. In-situ Raman spectroscopy was performed on a Thermo Scientific DXR3xi Raman microscope with a 532 nm laser wavelength. Raman spectra were acquired in a custom-designed electrochemical cell, containing the sample as the working electrode, a Hg/HgO reference electrode (1.0 M KOH), and a carbon rod counter electrode. The laser power was limited to ~2 mW to avoid degradation of the sample.

***Electrochemical measurements***

All electrochemical measurements were carried out in oxygen-saturated 1.0 M KOH at 25 ^o^C using a VersaSTAT 4 potentiostat (Princeton Applied Research). A standard three-electrode configuration was employed, consisting of the catalyst-coated Ni foam (1×1 cm^2^ geometric area) as the working electrode, a Hg/HgO (1.0 M KOH filing solution) reference electrode, and a graphite rod counter electrode. The polarization curves were recorded by cyclic voltammetry (CV) at a scan rate of 5 mV s^–1^. To minimize contributions from capacitive currents associated with Ni redox transitions, OER and overall water-splitting activities were determined from the backward sweep of CV curves. All measurements were repeated at least three times for reproducibility. The measured potentials (versus the Hg/HgO) were corrected for ohmic losses (iR compensation) using the uncompensated solution resistance (R_s_) obtained from electrochemical impedance spectroscopy (EIS), and then converted to the reversible hydrogen electrode (RHE) scale according to:

E_RHE_ = E_Hg/HgO_ + 0.098 + 0.059 × pH (at 25 ^o^C) (1)

The Tafel slope was determined from the linear portion of Tafel plots, according to:

𝜂 = *b*·log|*j*| + α (2)

where 𝜂 is the overpotential (V) defined as E_RHE_ − 1.23 V, *j* is the current density (mA cm^–2^), *b* is the Tafel slope (mV dec^–1^), and α is a constant.

Electrochemically active surface area (ECSA) was derived from the double-layer capacitance (C_dl_) measured through a series of CV at various scan rate in a non-Faradaic region:

C_dl_ = $\frac{\text{|}\text{j}_{\text{a}}\text{–}\text{ }\text{j}_{\text{c}}\text{|}}{\text{2}\text{v}}$ (3)

where *j*_a_ and *j*_c_ are the anodic and cathodic current densities at the midpoint of the potential window, respectively, and *v* is the scan rate.

The ECSA was calculated as:

ECSA = $\frac{\text{A·}\text{C}_{\text{dl}}}{\text{C}_{\text{s}}}$ (4)

where A is the geometric surface area (cm^2^) and C_s_ is the specific capacitance of bare Ni foam (0.6 mF cm^–2^)^[[2]](#endnote-2)^.

The intrinsic activity of catalysts was assessed by turnover frequency (TOF), calculated as:

TOF = $\frac{\text{A·}\text{j}}{\text{4·F·m}}$ (5)

where A is the geometric surface area (cm^2^) of the electrode, *j* is the current density (A cm^–2^), F is the Faraday constant (96,485 C mol^–1^), and m is molar quantity of electrochemically active sites (mol). The factor of 4 accounts for the number of electrons required per O_2_ molecule evolved.

The mass activity (MA) (A g⁻^1^) at a given overpotential was obtained by:

MA = $\frac{\text{j}}{\text{m}_{\text{s}}}$ (6)

​

where, *j* is the current density (A cm⁻^2^), and m_s_ is the total catalyst mass on the electrode (g cm^–2^).

The number of active sites (m) was estimated from CV data by analyzing the linear dependence of anodic peak current (i_a_) on scan rate (v), assuming a one-electron redox process (n = 1):

Slope = $\frac{\text{n}^{\text{2}}\text{·}\text{F}^{\text{2}}\text{·m}}{\text{4·R·T}}$ (7)

where R is the gas constant (8.314 J mol^–1^ K^–1^) and T is the absolute temperature (K).

To minimize the influence of surface reconstruction on active-site estimation, the electrodes were first electrochemically preconditioned by 10 CV cycles and the i_a_–ν measurements were conducted within a restricted potential window around the stable redox couple.

Operando EIS was carried out in the frequency range of 0.1 Hz–100 kHz using a 10 mV AC perturbation at various applied potentials. Stability tests were performed under galvanostatic conditions, including stepwise current increments from 50 to 400 mA cm^–2^ (50 mA cm^–2^ per step, 1 h per step), as well as extended continuous operation at 10 and 500 mA cm^–2^. Differential pulse voltammetry (DPV) measurements were carried out in 1.0 M KOH by scanning from 0.2 to 0.7 V (vs Hg/HgO), using a pulse amplitude of 30 mV, a pulse duration of 50 ms, a step potential of 5 mV, and a step width of 0.5 s.

**Supporting tables**

**Table S1.** Comparison of OER activities (in 1.0 M KOH) for various high-performing transition metal-base electrocatalysts.

| **Catalyst** | **η_10_ (mV)** | **Tafel slope**  **(mV dec^–1^)** | **Reference** |
| --- | --- | --- | --- |
| **FeOOH@Ni_3_N/NF** | **209**  **245 (**η_100_**)** | **38.9** | **This work** |
| N-NiMoO_4_/NiS_2_ | 283 | 44 | *Adv. Funct. Mater.* **2019**, 29, 1805298. |
| FeOOH(Se)/IF | 287 | 54 | *J. Am. Chem. Soc.* **2019**, 141, 7005. |
| Zn_0.2_Co_0.8_OOH | 235 | 35.7 | *Nat. Energy* **2019**, 4, 329. |
| CoNiLDH/FeOOH | 250 | 60 | *Small* **2023**, 19, 2305241. |
| Ni–Ni_3_S_2_/NF | 295 (η_20_) | 99 | *Adv. Energy Sustainability Res.* **2021**, 2, 2100078. |
| Co_3_O_4_/Co-Fe oxide | 297 | 61 | *Adv. Mater.* **2018**, 30, 1801211. |
| Co-Ni_3_N | 307 | 57 | *Adv. Mater.* **2018**, 30, 1705516. |
| NiTe/NiS | 244 | 49 | *Adv. Mater.* **2019**, 31, 1900430. |
| N-doped CNTFs | 370 | 93 | *Nat. Energy* **2016**, 1, 15006. |
| Ni_0.5_Co_0.5_-MOF-74 | 265 | 49 | *Nat. Energy* **2020**, 5, 881. |
| W-Ni(OH)_2_ | 237 | 33 | *Nat. Commun.* **2019**, 10, 2149. |
| P-Co_3_O_4_ | 280 | 51.6 | *Energy Environ. Sci.* **2017**, 10, 2563. |
| δ-FeOOH NSs/NF | 265 | 69 | *Adv. Mater.* **2018**, 30 1803144. |
| Mn-N-doped graphene | 337 | 55 | *Nat. Catal.* **2018**, 1, 870. |
| Co_80_Fe_20_(OH)(OCH_3_) | 240 | 53.4 | *Adv. Funct. Mater.* **2021**, 31, 2009245. |
| NiCo_2-x_Fe_x_O_4_ NBs | 274 | 42 | *Angew. Chem. Int. Ed.* **2021**, 60, 11841. |
| Ni_3_FeN@PO_4_^3−^/NF | 228 | 29.9 | *Adv. Mater.* **2024**, 2415421. |
| Ir_1_/V_O_–CoOOH | 200 | 32 | *Nat. Commun.* **2022**, 13, 2473. |
| NiMoO_x_/NiMoS | 186 | 34 | *Nat. Commun.* **2020**, 11, 5462. |
| Ni_83_Fe_17_-ONCAs | 190 | 39 | *Adv. Mater.* **2021**, 33, 2007377. |
| Co_1.8_Ni(OH)_5.6_ | 274 | 45 | *Adv. Mater.* **2019**, 31, 1805658. |
| Porous Ni_3_S_4_ | 257 | 67 | *Adv. Funct. Mater.* **2019**, 29, 1900315. |
| Ni_0.78_Fe_0.22_-LDH | 243 | 55 | *Mater. Horiz.* **2023**, 10, 632. |
| Au-NiFeOOH | 237 | 36 | *J. Am. Chem. Soc.* **2018**, 140, 3876. |
| Amorphous CoS_4.6_O_0.6_ | 290 | 67 | *Angew. Chem. Int. Ed.* **2017***,* 56, 4858. |
| NiCo-UMOFNs | 250 | 142 | *Nat. Energy*, **2016**, 1, 16181. |
| Ultrathin FeCo LDH | 232 | 36 | *Adv. Mater.* 2017, 29, 1701546. |
| FeOOH/Co/FeOOH | 240 | 32 | *Angew. Chem. Int. Ed.* **2016**, 55, 3694. |
| NiFe LDH/GO | 210 | 40 | *ACS Nano* **2015**, 9, 1977. |
| NiCo-UMOFNs | 250 | 42 | *Adv. Mater.* **2021**, 33, 2006042. |
| NiFe-MOF | 240 | 34 |  |

**Table S2.** Comparison of the overall water splitting activity data (at 10 mA cm^–2^) of various recently-reported high-performance electrocatalysts.

| **Catalyst** | **Potential (V)** | **Reference** |
| --- | --- | --- |
| **FeOOH@Ni_3_N/NF** | **1.49@10 mA/cm^2^** | **This work** |
| Ni-Fe NPs | 1.47 | *Nat. Commun.* **2019***,* 10, 5599. |
| NiFeO_x_/CNF | 1.51 | *Nat. Commun.* **2015***,* 6, 7261. |
| IFONFs | 1.58 | *Nat. Commun.* **2018***,* 9, 1809. |
| RuIrOx | 1.47 | *Nat. Commun.* **2019***,* 10, 4875. |
| Ni_3_S_2_ NSs | 1.76 | *J. Am. Chem. Soc.* **2015***,* 137, 14023. |
| NiFe-NiMo/Ni-P | 1.51 | *Nat. Commun.* **2018***,* 9, 2014. |
| R-NiCo_2_O_4_ | 1.61 | *J. Am. Chem. Soc.* **2018***,* 140, 13644. |
| MoO_3_/Ni-NiO | 1.55 | *Adv. Mater.* **2020***,* 32, 2003414. |
| W_2_N/WC | 1.58 | *Adv. Mater.* **2020***,* 32, 1905679. |
| NiFe MOF | 1.55 | *Nat. Commun.* **2017***,* 8, 15341. |
| CoP/NCNHP | 1.64 | *J. Am. Chem. Soc.* **2018***,* 140, 2610. |
| Co/CNFs | 1.69 | *Adv. Mater.* **2019***,* 31, 1808043. |
| CoMoNiS-NF | 1.54 | *J. Am. Chem. Soc.* **2019***,* 141, 10417. |
| Fe_0.09_Co_0.13_-NiSe_2_ | 1.52 | *Adv. Mater.* **2018***,* 30, 1802121. |
| Co_2_P/CoNPC | 1.64 | *Adv. Mater*. **2020**, 32, 2003649. |
| Co_3_S_4_/EC-MOF | 1.55 | *Adv. Mater.* **2019***,* 31, 1806672. |
| δ-FeOOH NSs/NF | 1.62 | *Adv. Mater*. **2018**, 30 1803144. |
| Cr-FeNi-P/NCN | 1.50 | *Adv. Mater.* **2019***,* 31, 1900178. |
| CoP NC | 1.56 | *Adv. Mater.* **2018***,* 30, 1705796. |
| CoSn_2_ | 1.55 | *Angew. Chem. Int. Ed.* **2018***,* 57, 15237. |
| SrCo_0.85_Fe_0.1_P_0.05_O_3-δ_ | 1.63 | *Adv. Mater.* **2018***,* 30, 1804333. |
| Ir1@Co/NC | 1.60 | *Angew. Chem. Int. Ed.* **2019***,* 58, 11868. |
| Ni/Mo_2_C | 1.64 | *Adv. Energy Mater.* **2019***,* 9, 1803185. |
| RuCu NSs/C | 1.49 | *Angew. Chem. Int. Ed.* **2019***,* 58, 13983. |
| Pt/LiCoO_2_ | 1.54 | *Angew. Chem. Int. Ed.* **2020***,* 59, 14533. |
| Pt-CoS_2_/CC | 1.55 | *Adv. Energy Mater.* **2018***,* 8, 1800935. |
| MoNi_4_/SSW | 1.87@100 mA/cm^2^ | *Adv. Energy Mater.* **2020**, 10, 1904020. |
| NC/NiCu/NiCuN | 1.56 | *Adv. Funct. Mater.* **2018**, 28, 1803278. |
| NC/CuCo/CuCoOx | 1.53 | *Adv. Funct. Mater.* **2018***,* 28, 1704447. |
| P-Co_3_O_4_/NF | 1.63 | *ACS Catal.* **2018**, 8, 2236. |
| Mo_2_S_3_@NiMo_3_S_4_ | 1.56@100 mA/cm^2^ | *Adv. Sci.* **2022**, 9, 2202750. |
| Ni_2_P-Fe_2_P/NF | 1.56 | *Adv. Funct. Mater.* **2021**, 31, 2006484. |
| Ni_0.8_Fe_0.2_-AHNAs | 1.41 | *Energ. Environ. Sci.* **2020**, 13, 86. |
| Co_4_N-CeO_2_ | 1.51 | *Adv. Funct. Mater.* **2020**, 30, 1910596. |
| NiMoN@NiFeN | 1.56@100 mA/cm^2^ | *Nat. Commun.* **2019**, 10, 5106. |
| FeP/Ni_2_P | 1.42 | *Nat. Commun.* **2018**, 9, 2551 |
| NiMoOx/NiMoS | 1.46 | *Nat. Commun.* **2020**, 11, 5462. |
| CoMoS_x_/NF | 1.74@100 mA/cm^2^ | *Angew. Chem. Int. Ed.* **2020**, 59, 1659. |

**Table S3.** Comparison of the overall water splitting activity data (at 500 mA cm^–2^) for various recently-reported high-performance electrocatalysts.

| **Catalyst** | **Potential (V)** | **Reference** |
| --- | --- | --- |
| **FeOOH@Ni_3_N/NF** | **1.72** | **This work** |
| NiMoO_x_/NiMoS | 1.75 | *Nat. Commun.* **2020**, 11, 5462. |
| NiFe-LDH/MXene/NF | 1.75 | *Nano Energy* **2019**, 63, 103880. |
| CoMoS_x_/NF | 1.89 | *Angew. Chem. Int. Ed.* **2020***,* 59, 1659. |
| Pt_SA_-Mn,Fe-Ni LDH | 1.79 | *ACS. Nano.* **2024**, 18, 16222-35. |
| B,V-doped Ni_2_P | 1.78 | *Small* **2023**, 19, 2208076. |
| CoVO@NF | 1.76 | *Adv. Mater.* **2024**, *36*, 2408634. |
| Co_4_N-CeO_2_ | 1.99 | *Adv. Funct. Mater.* **2020**, 30, 1910596. |
| P-doped Os | 1.86 | *Adv. Funct. Mater.* **2024**, *34*, 2408517. |
| NiSe_2_/NiFe_2_Se_4_@NiFe | 1.96 | *Nano-Micro Lett.* **2020**, 12, 104. |
| WO_2_-Ni_17_W_3_)/NiFe(OH)_x_/NF | 1.75 | *Chem. Eng. J.* **2022**, 431, 134247. |
| FeCo/Ni(OH)_2_/Ni mesh | 1.91 | *Int. J. Hydrog. Energy* **2023**, 48, 17882. |
| Ni_3_P/MnOOH | 2.00 | *Electrochim. Acta* **2019**, 324, 134897. |
| rNi-FeNi_3_ | 1.85 | *Appl. Catal. B* **2021**, 286, 119881. |
| Mn_0.15_-NiFe LDH/Fe_0.64_Ni_0.36_/NM | 1.78 | *Nano Res.* **2023**, 16, 8953. |
| V–Ni_2_P/Ni_12_P_5_ | 1.79 | *Small* **2022**, 18, 2204758. |
| Ir_SA_-NiFe LDH/NiMo | 1.85 | *Appl. Catal. B* **2025**, 360, 124548. |

**Table S4.** EIS fitting parameters for FeOOH@Ni_3_N/NF and Ni_3_N/NF electrocatalysts using the Armstrong–Henderson equivalent circuit model.

| **Ni_3_N/NF** | | | | | | | | |
| --- | --- | --- | --- | --- | --- | --- | --- | --- |
| **Potential**  **(V vs RHE)** | **R_s_**  **(Ω)** | **CPE_dl_**  **(mF∙s^a1-1^)** | **a_1_** | **R_ct_**  **(Ω)** | **CPE_ad_**  **(mF∙s^a2-1^)** | **a_2_** | **R_i_**  **(Ω)** | **x^2^** |
| 1.25 | 0.86 | 6.5 | 0.84 | 16.62 | 2.8 | 0.96 | - | 1.5×10^-3^ |
| 1.30 | 0.88 | 8.5 | 0.83 | 16.63 | 4.0 | 1 | - | 1.7×10^-3^ |
| 1.35 | 0.86 | 14.3 | 0.77 | 17.76 | 6.9 | 0.92 | - | 5.4×10^-3^ |
| 1.40 | 0.86 | 25.6 | 0.73 | 13.31 | 10.3 | 1 | - | 5.1×10^-3^ |
| 1.45 | 0.90 | 29.9 | 0.84 | 17.46 | 20.5 | 1 | 312.04 | 4.1×10^-4^ |
| 1.50 | 0.89 | 28.6 | 0.84 | 10.01 | 33.3 | 1 | 12.16 | 1.4×10^-4^ |
| 1.55 | 0.88 | 30.4 | 0.83 | 1.61 | 71.0 | 1 | 2.17 | 3.7×10^-4^ |
| 1.60 | 0.88 | 28.2 | 0.83 | 0.48 | 108.4 | 1 | 0.52 | 1.9×10^-4^ |
| **FeOOH@Ni_3_N/NF** | | | | | | | | |
| **Potential**  **(V vs RHE)** | **R_s_**  **(Ω)** | **CPE_dl_**  **(mF∙s^a1-1^)** | **a_1_** | **R_ct_**  **(Ω)** | **CPE_ad_**  **(mF∙s^a2-1^)** | **a_2_** | **R_i_**  **(Ω)** | **x^2^** |
| 1.25 | 0.92 | 12.0 | 0.78 | 8.96 | 26.1 | 0.59 | - | 8.4 × 10^-4^ |
| 1.30 | 0.92 | 20.1 | 0.74 | 11.82 | 28.2 | 0.63 | - | 3.2 × 10^-4^ |
| 1.35 | 0.92 | 31.2 | 0.72 | 16.75 | 29.4 | 0.95 | - | 5.0 × 10^-4^ |
| 1.40 | 0.91 | 41.0 | 0.71 | 13.67 | 61.3 | 0.98 | - | 8.5 × 10^-4^ |
| 1.45 | 0.90 | 117.3 | 0.85 | 0.16 | 163.7 | 0.82 | 4.05 | 4.1 × 10^-4^ |
| 1.50 | 0.91 | 107.0 | 0.87 | 0.12 | 213.5 | 0.88 | 0.63 | 1.4 × 10^-4^ |
| 1.55 | 0.91 | 105.8 | 0.85 | 0.10 | 232.3 | 0.91 | 0.25 | 5.5 × 10^-4^ |
| 1.60 | 0.91 | 117.2 | 0.83 | 0.09 | 253.7 | 0.91 | 0.14 | 4.6 × 10^-4^ |

**Supporting figures**


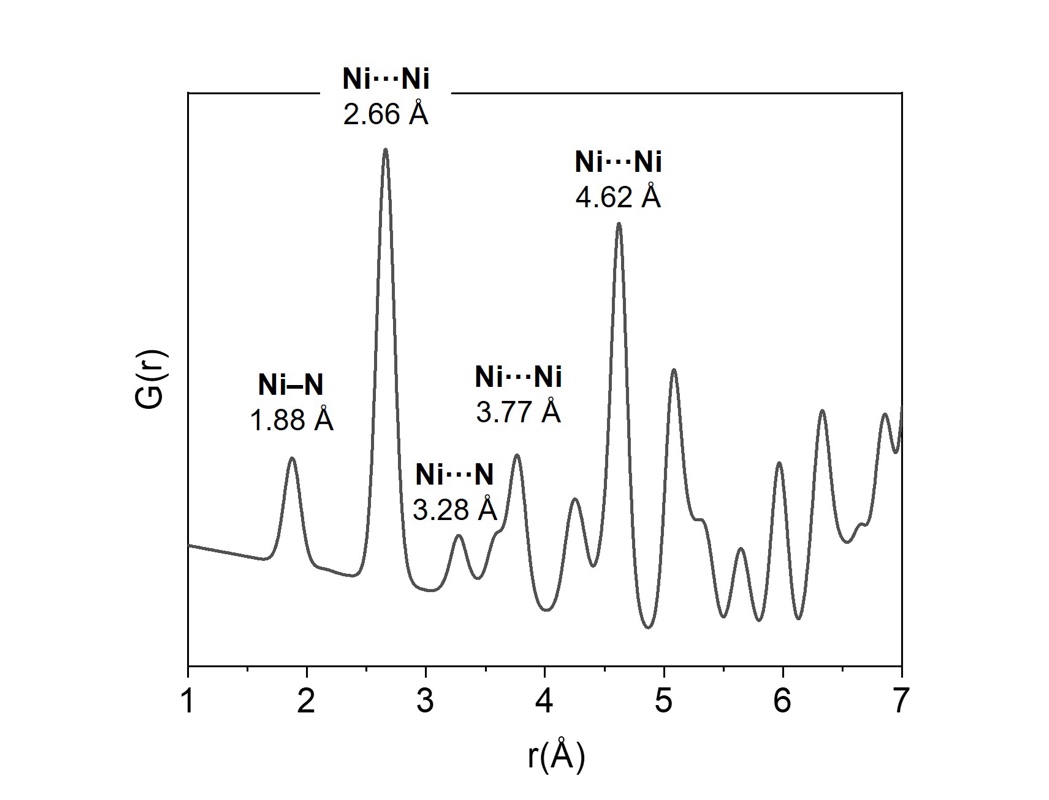


**Figure S1**. Calculated PDF plot of hexagonal Ni_3_N (space group *P*6_3_22; JCPDS card no. 89-5144).


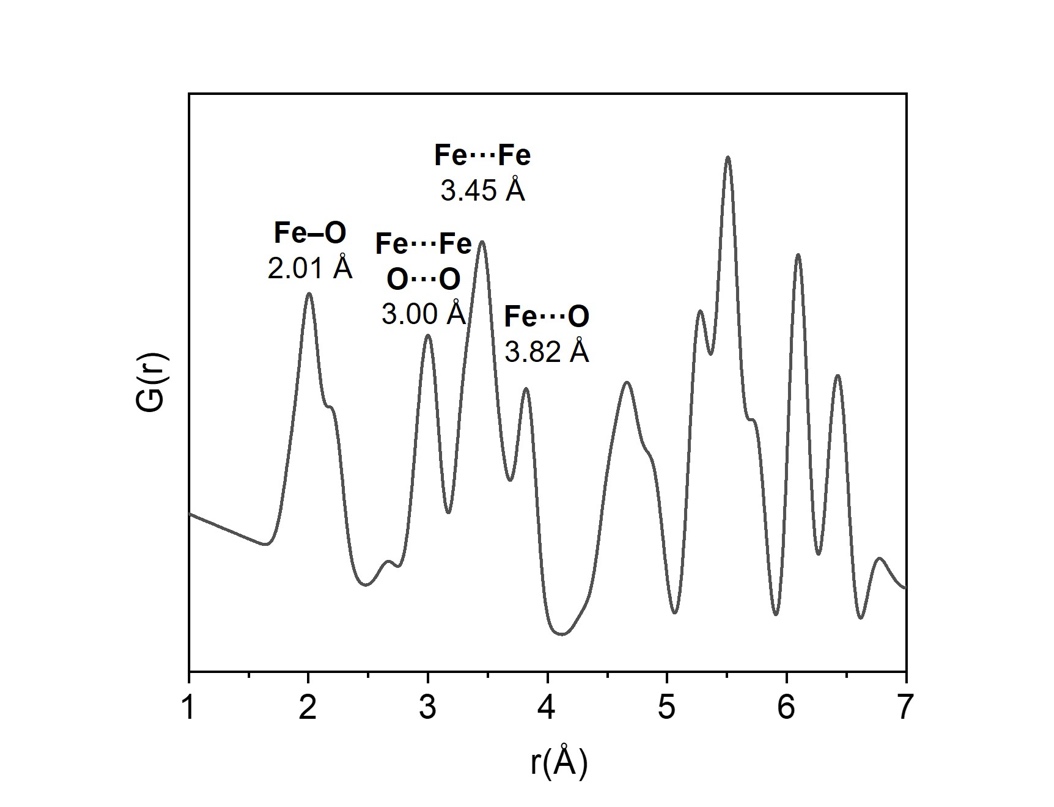


**Figure S2**. Calculated PDF plot of orthorhombic α-FeOOH (space group *Pnma*; JCPDS card no. 73-6522).

**
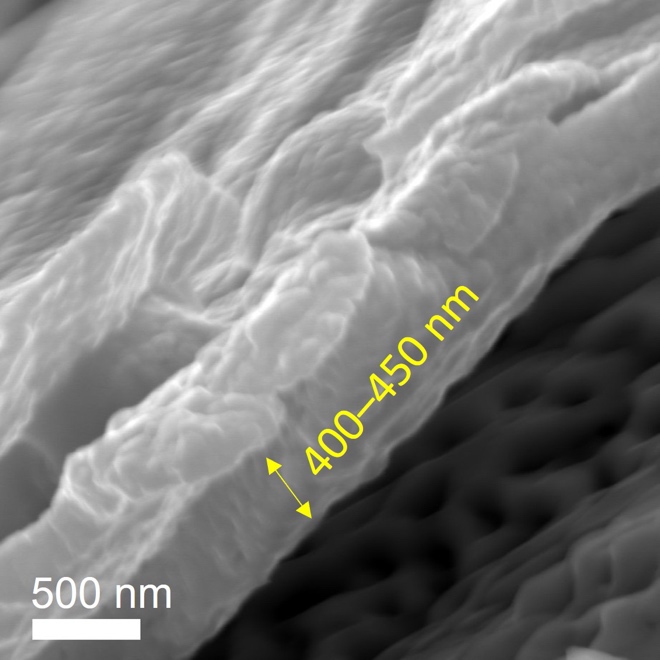
**

**Figure S3**. Cross-sectional SEM image of Ni_3_N/NF, showing a uniform Ni_3_N layer with an average thickness of approximately 400–450 nm on the Ni foam substrate.


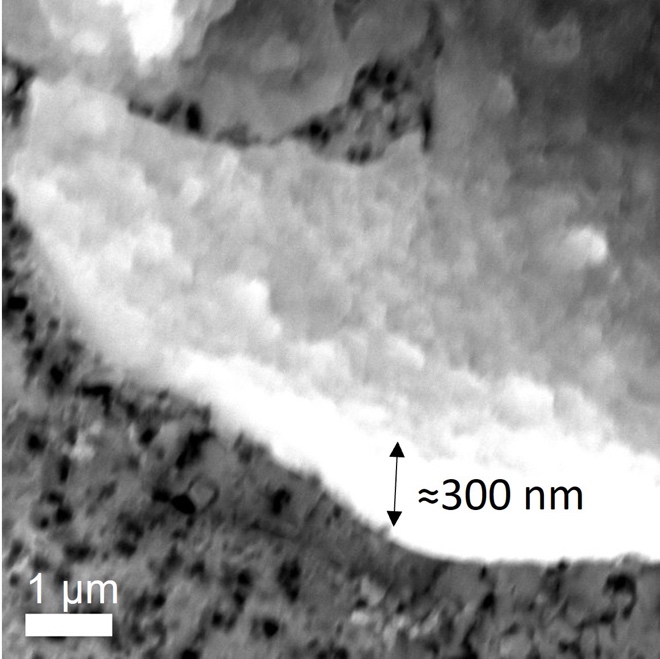


**Figure S4**. Cross-sectional SEM image of FeOOH@Ni_3_N/NF, showing a α-FeOOH overlayer with an average thickness of approximately 300 nm.


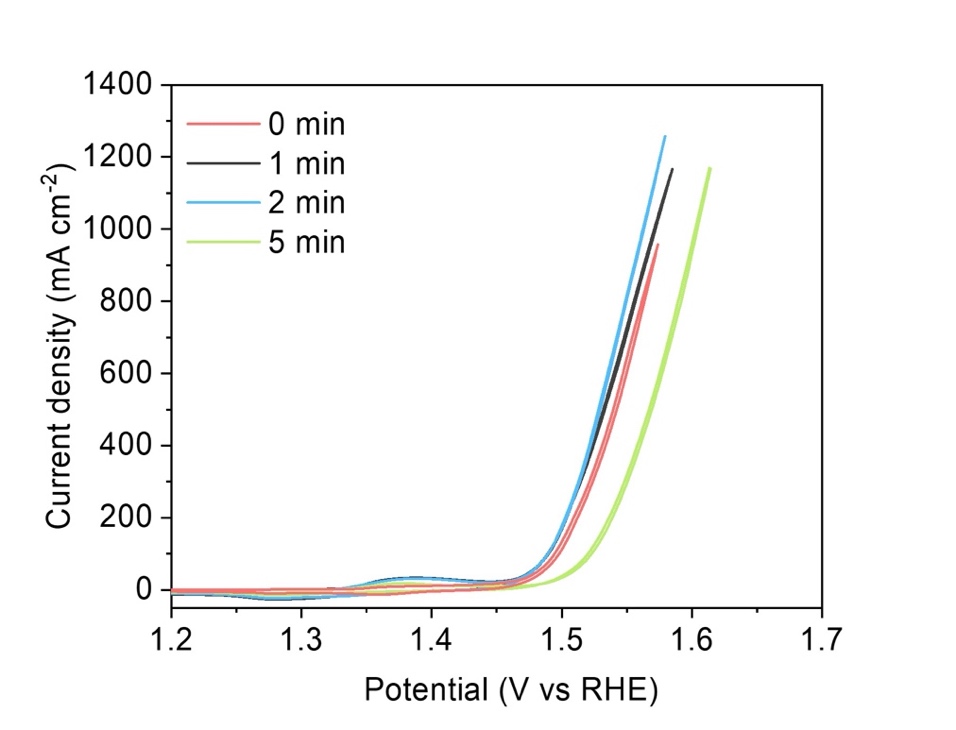


**Figure S5.** iR-corrected OER polarization curves in recorded 1.0 M KOH (scan rate: 5 mV s^–1^) for FeOOH@Ni_3_N/NF electrocatalysts synthesized with different impregnation times in 15 mM Ni(NO_3_)_2_ solution, followed by nitridation at 500 ^o^C for 1 h and subsequent 10 min impregnation in 15 mM Fe(NO_3_)_3_ solution.


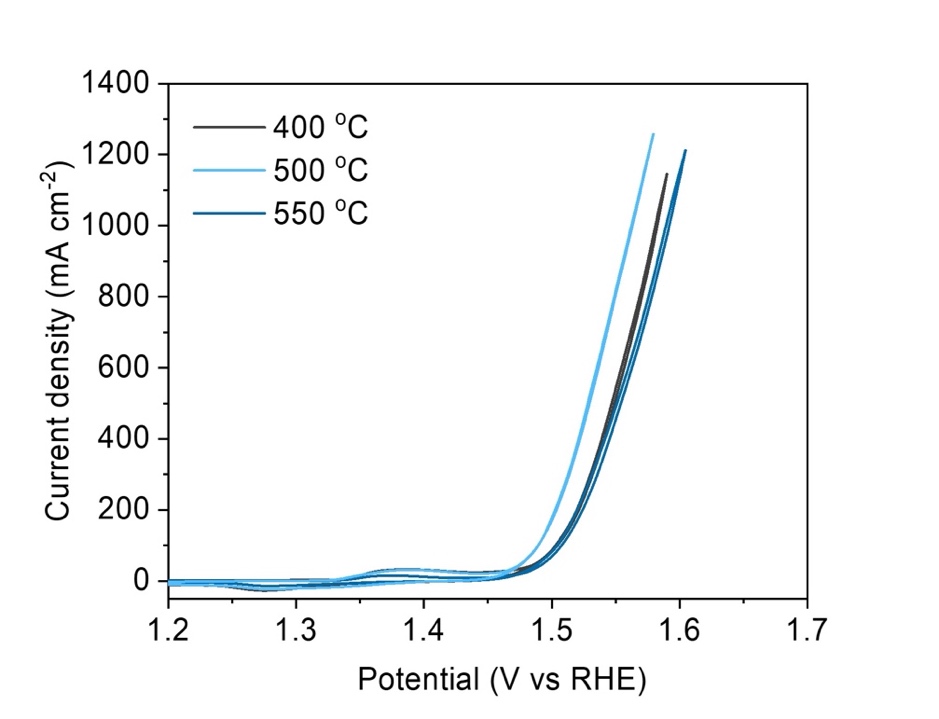


**Figure S6.** iR-corrected OER polarization curves recorded in 1.0 M KOH (scan rate: 5 mV s^–1^) for FeOOH@Ni_3_N/NF electrocatalysts synthesized at different nitridation temperatures (1 h duration). Synthesis conditions: 2 min immersion of Ni foam in 15 mM Ni(NO_3_)_2_ solution, followed by 10 min impregnation in 15 mM Fe(NO_3_)_3_ solution after the respective nitridation step.


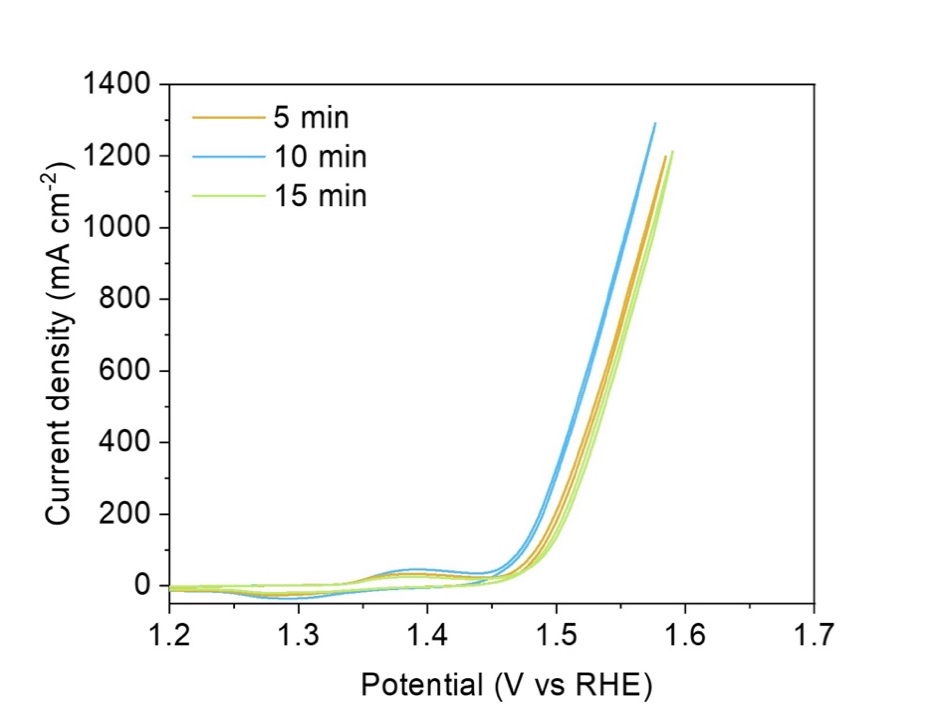


**Figure S7.** iR-corrected OER polarization curves recorded in 1.0 M KOH (scan rate: 5 mV s^–1^) for FeOOH@Ni_3_N/NF electrocatalysts synthesized with different impregnation times in 15 mM Fe(NO_3_)_3_ solution. Synthesis conditions: 2 min immersion of Ni foam in 15 mM Ni(NO_3_)_2_ solution, followed by nitridation at 500 ^o^C.


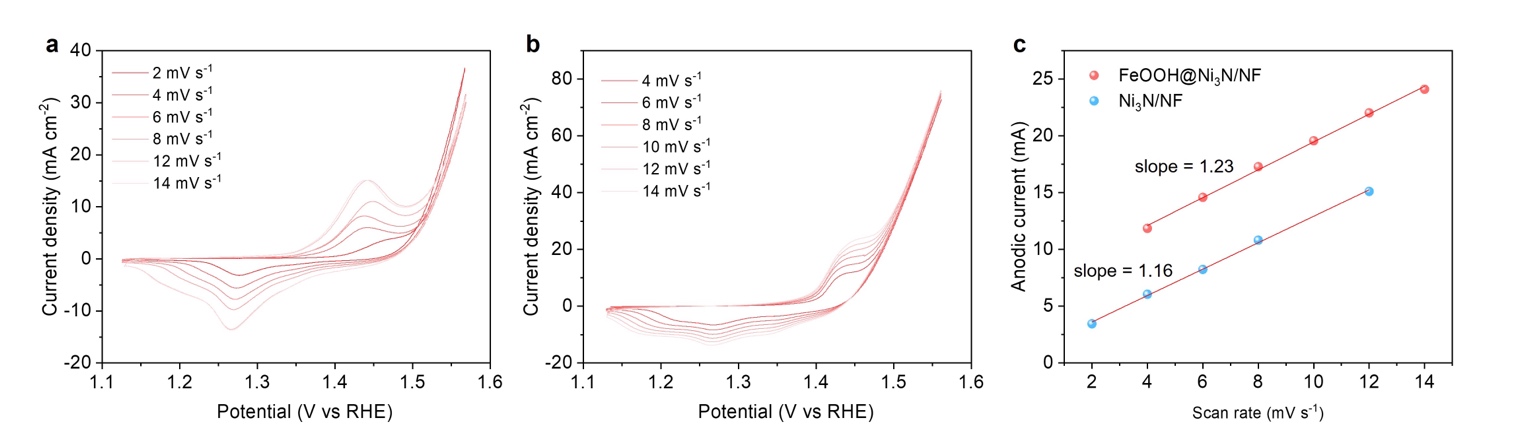


**Figure S8.** Cyclic voltammetry (CV) curves of (a) Ni_3_N/NF and (b) FeOOH@Ni_3_N/NF recorded at scan rates ranging from 2 to 14 mV s^–1^ in 1.0 M KOH electrolyte. (c) Corresponding plots of oxidation peak current versus scan rate, yielding slopes of 1.16 ± 0.02 and 1.23 ± 0.03 for Ni_3_N/NF and FeOOH@Ni_3_N/NF, respectively.


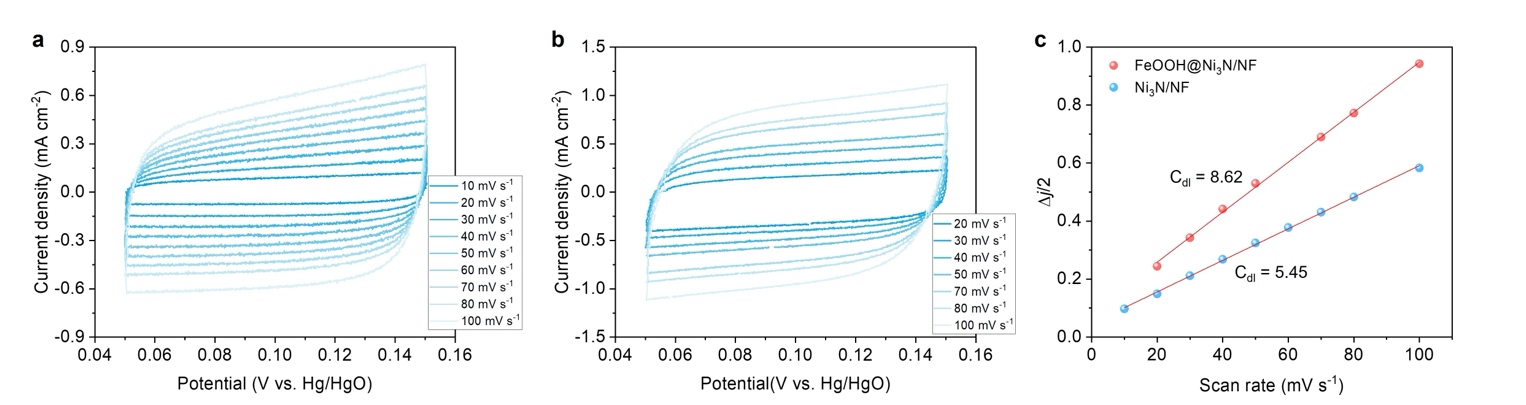


**Figure S9.** Cyclic voltammetry (CV) curves of (a) Ni_3_N/NF and (b) FeOOH@Ni_3_N/NF recorded in a non-Faradaic potential window (0.05–0.15 V vs. Hg/HgO) at scan rates ranging from 10 to 120 mV s^–1^. (d) Plots of Δ*j*/2 = (|*j*_a_ – *j*_c_|)/2 at 0.1 V vs Hg/HgO as a function of scan rate, used to extract the double-layer capacitance (C_dl_) for estimating the electrochemically active surface area (ECSA) of Ni_3_N/NF and FeOOH@Ni_3_N/NF electrocatalysts.


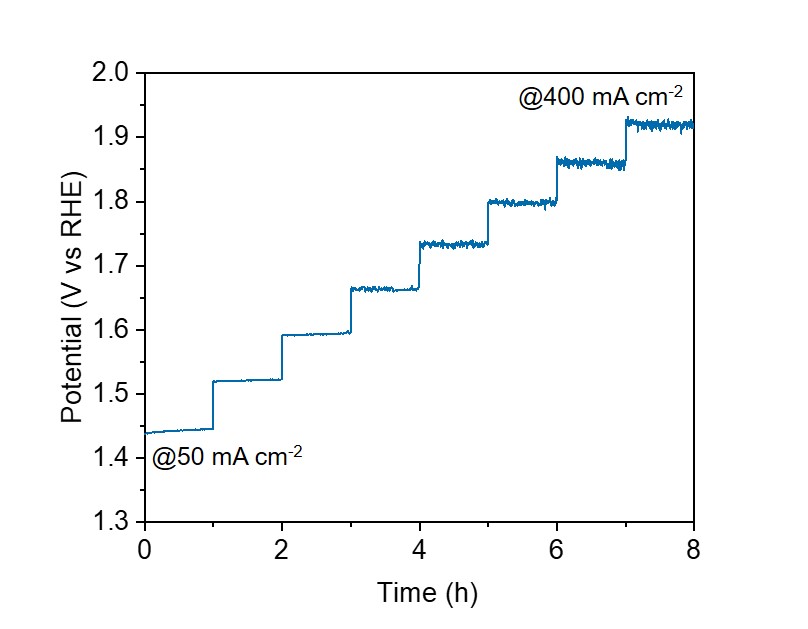


**Figure S10.** Multi-step chronopotentiometry test of FeOOH@Ni_3_N/NF performed in 1.0 M KOH electrolyte (without iR compensation), featuring sequential 1-h intervals with 50 mA cm^−2^ stepwise current density increments from 50 to 400 mA cm^−2^, demonstrating stable potential response and excellent operational durability under progressively increasing OER loads.


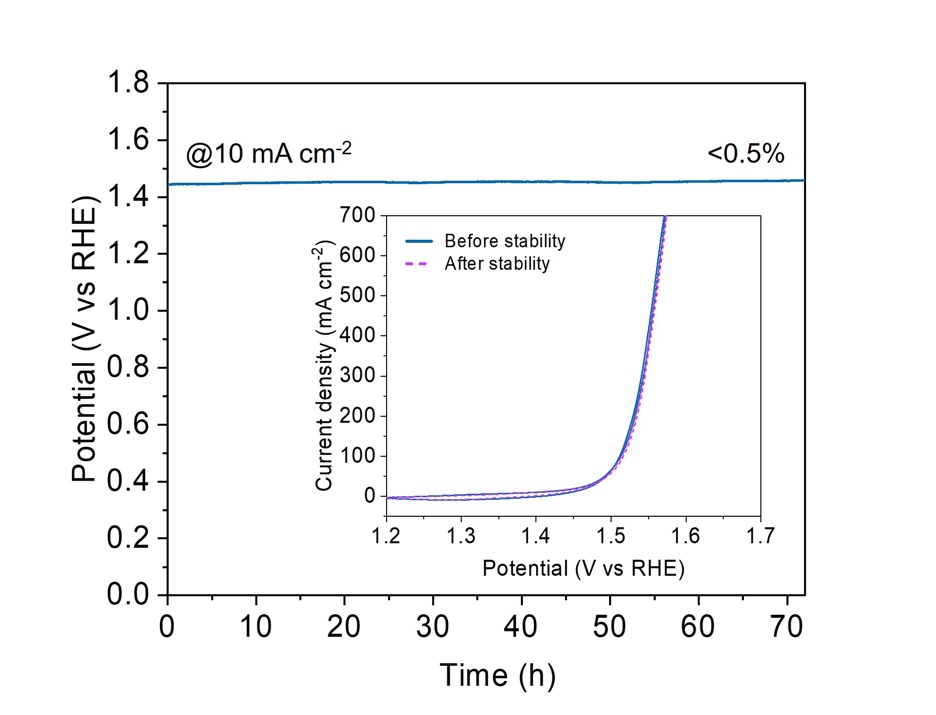


**Figure S11.** Galvanostatic stability test of FeOOH@Ni_3_N/NF conducted at a geometric current density of 10 mA cm^–2^ for 72 h in 1.0 M KOH. Inset: CV curves recorded before and after the durability test.


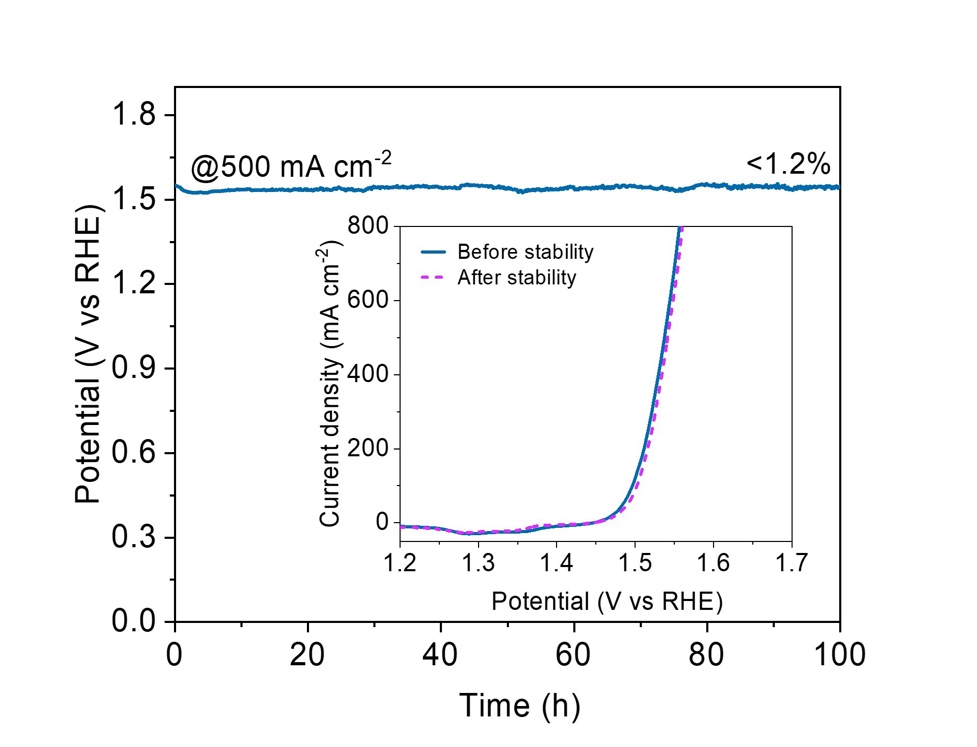


**Figure S12.** Galvanostatic stability test of FeOOH@Ni_3_N/NF conducted at a geometric current density of 500 mA cm^–2^ for 100 h in 1.0 M KOH. Inset: CV curves recorded before and after the durability test.


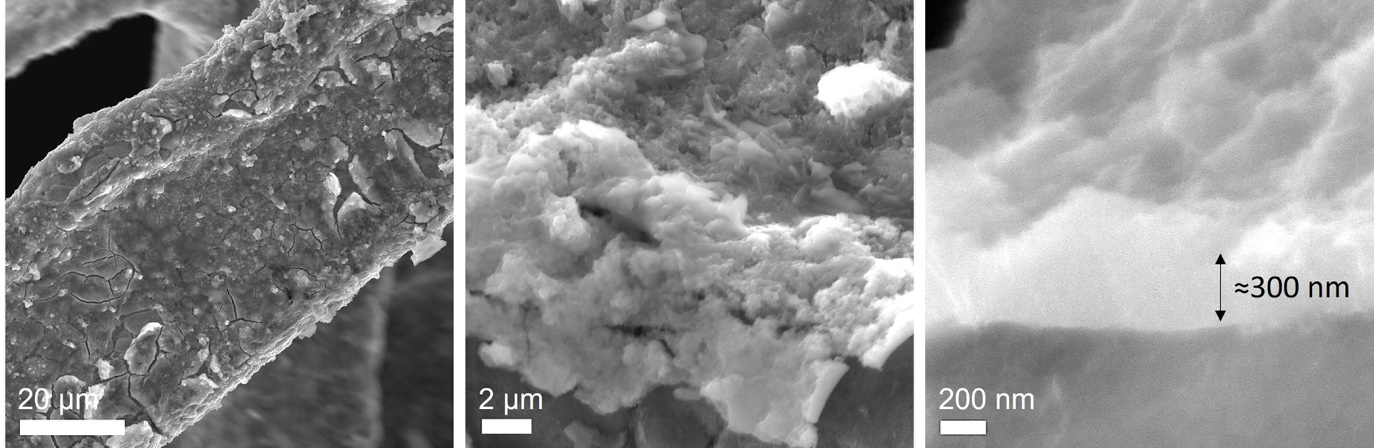


**Figure S13.** Representative SEM images of FeOOH@Ni_3_N/NF after high-current OER operation (500 mA cm^–2^ for 24 h), showing a wrinkled, cauliflower-like morphology of the α-FeOOH overlayer with an average thickness of ≈300 nm.


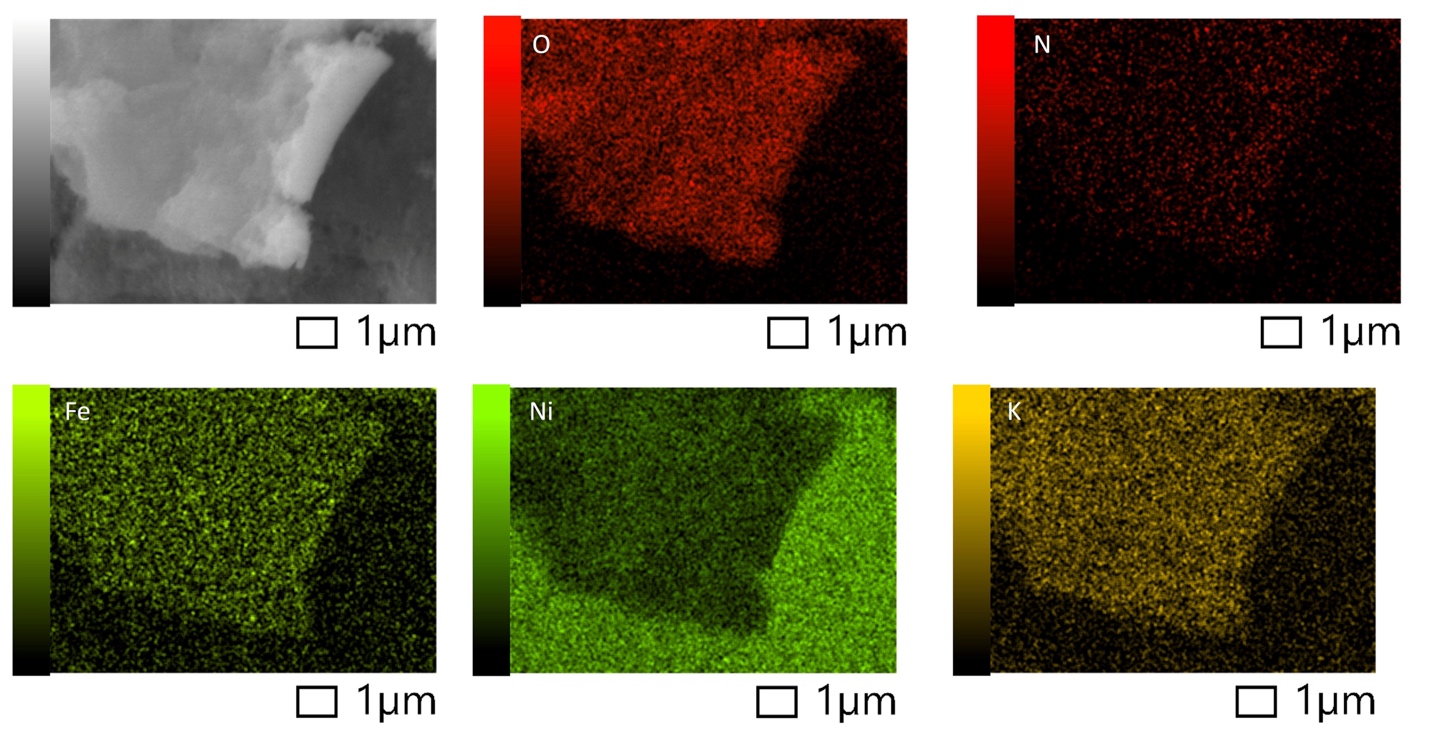


**Figure S14.** SEM-EDS elemental mapping of Fe, Ni, N, O and K for FeOOH@Ni_3_N/NF after 24 h of OER durability testing at 500 mA cm^–2^.

**
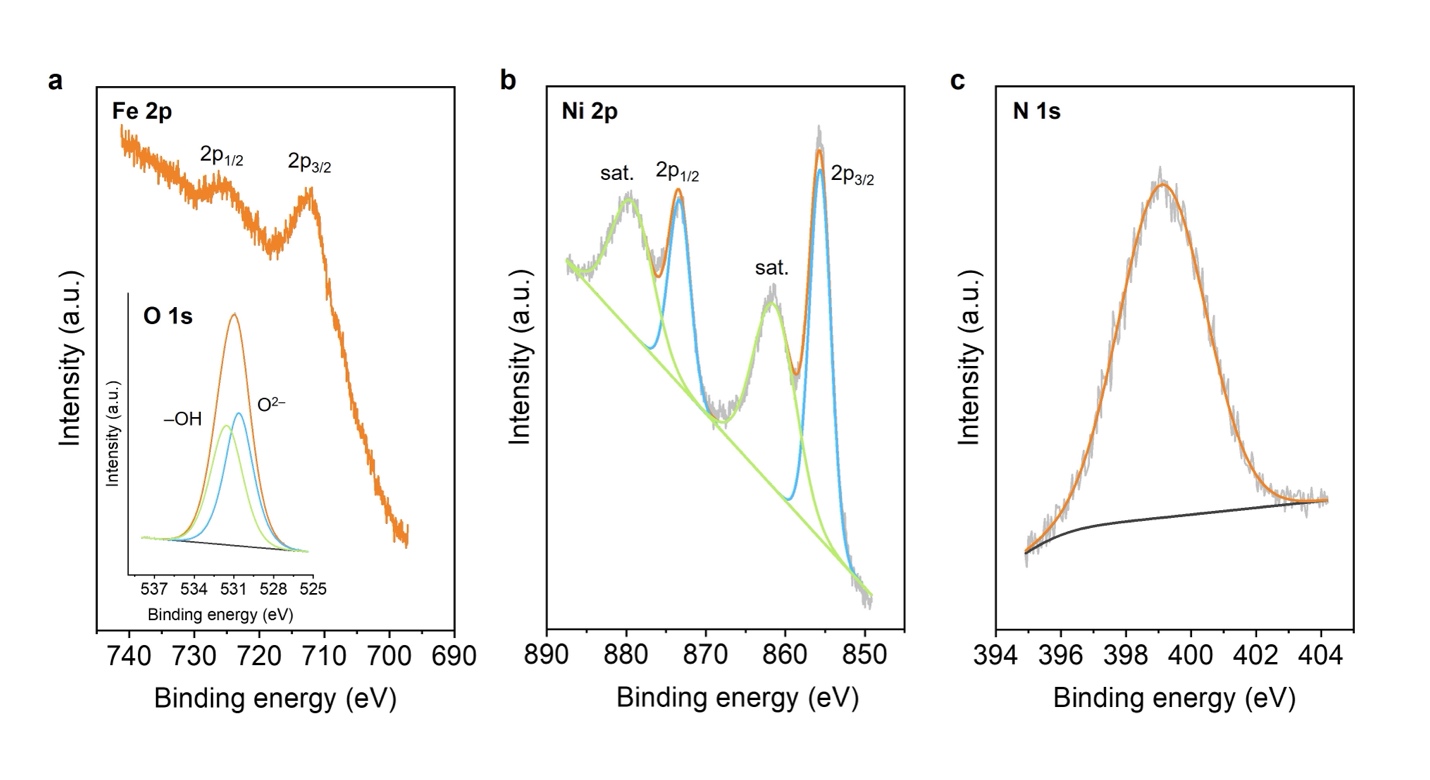
**

**Figure S15.** XPS spectra of (a) Fe 2*p* and O 1*s* (inset), (b) Ni 2*p* and (c) N 1*s* regions for FeOOH@Ni_3_N/NF after continuous OER operation for 24 h at 500 mA cm^–2^. Deconvoluted peaks assigned to distinct chemical species are shown in blue and green, while the orange curves represent the overall fitted envelopes.


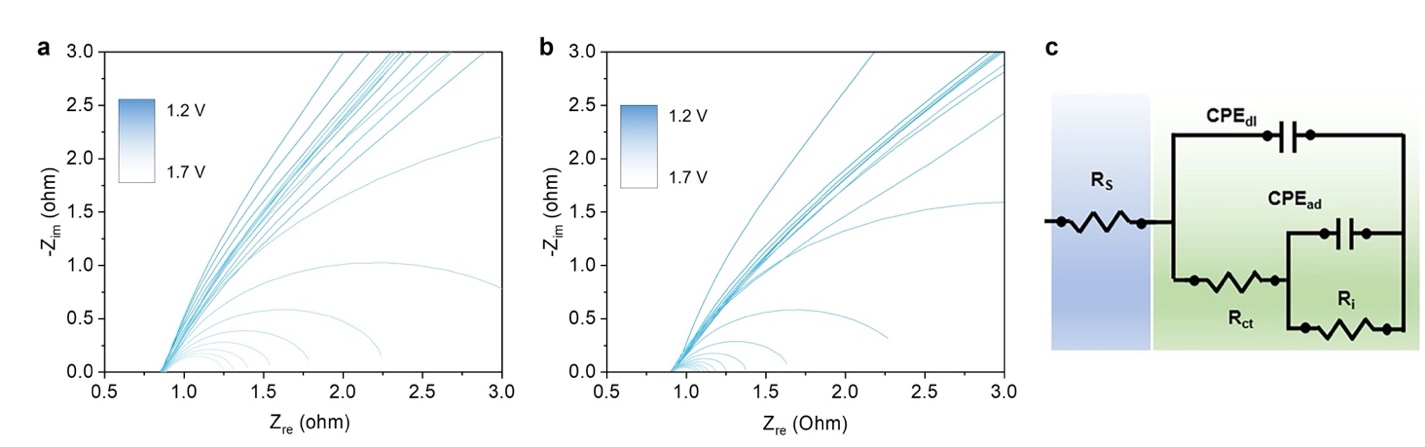


**Figure S16.** Operando electrochemical impedance spectroscopy (EIS) analysis of (a) Ni_3_N/NF and (b) FeOOH@Ni_3_N/NF electrocatalysts recorded at applied potentials ranging from 1.2 to 1.7 V vs RHE with 0.025 V increments. Nyquist plots were obtained over a frequency range of 100 kHz to 0.1 Hz. (**c**) The Armstrong-Henderson equivalent circuit used for fitting the EIS data, where R_s_ represents the solution resistance, R_ct_ denotes the interface reaction charge-transfer resistance and CPE_ad_ accounts for the capacitance response associated with adsorbed intermediates.


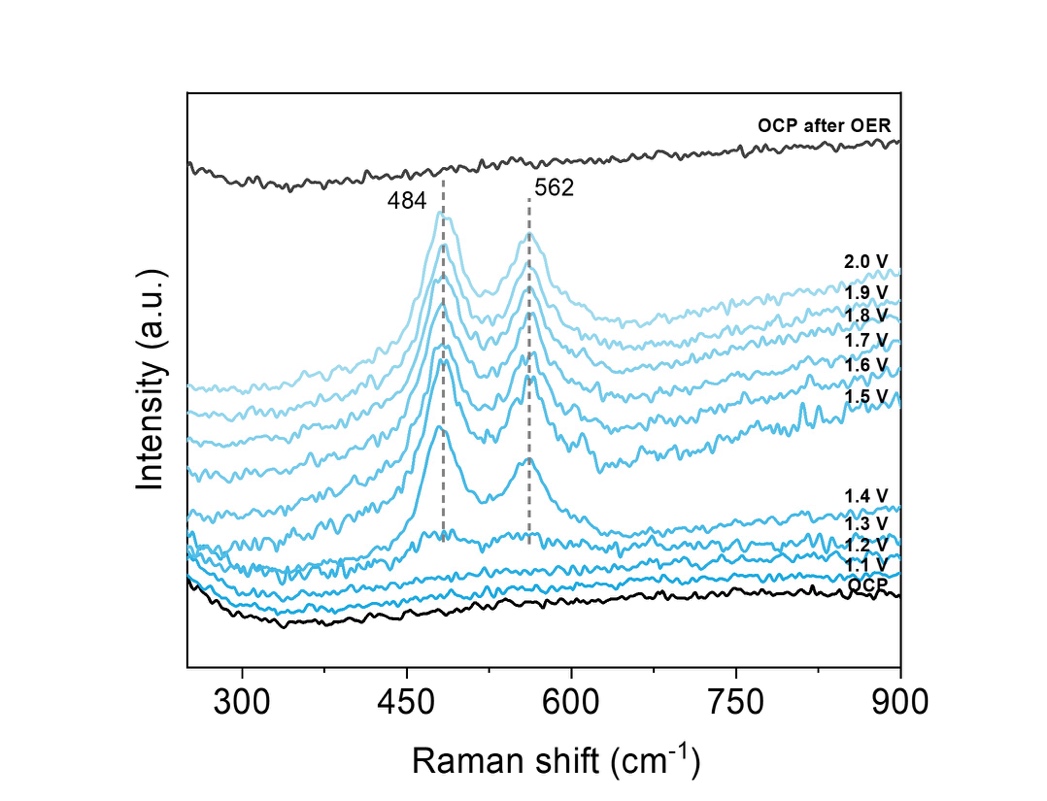


**Figure S17.** In situ Raman spectra of Ni_3_N/NF recorded during OER from open-circuit potential (OPC) to 2.0 V vs RHE in 1.0 M KOH.


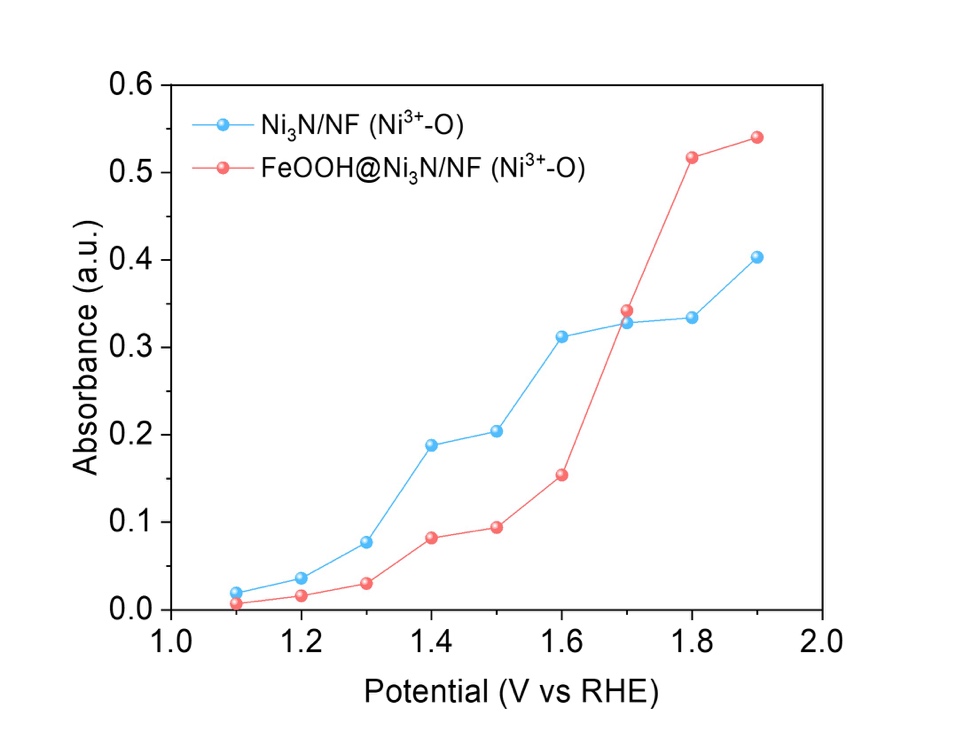


**Figure S18.** Potential-induced evolution of the Ni^3+^–O species (365–370 nm UV-vis absorbance) for Ni_3_N/NF and FeOOH@Ni_3_N/NF electrocatalysts during OER.


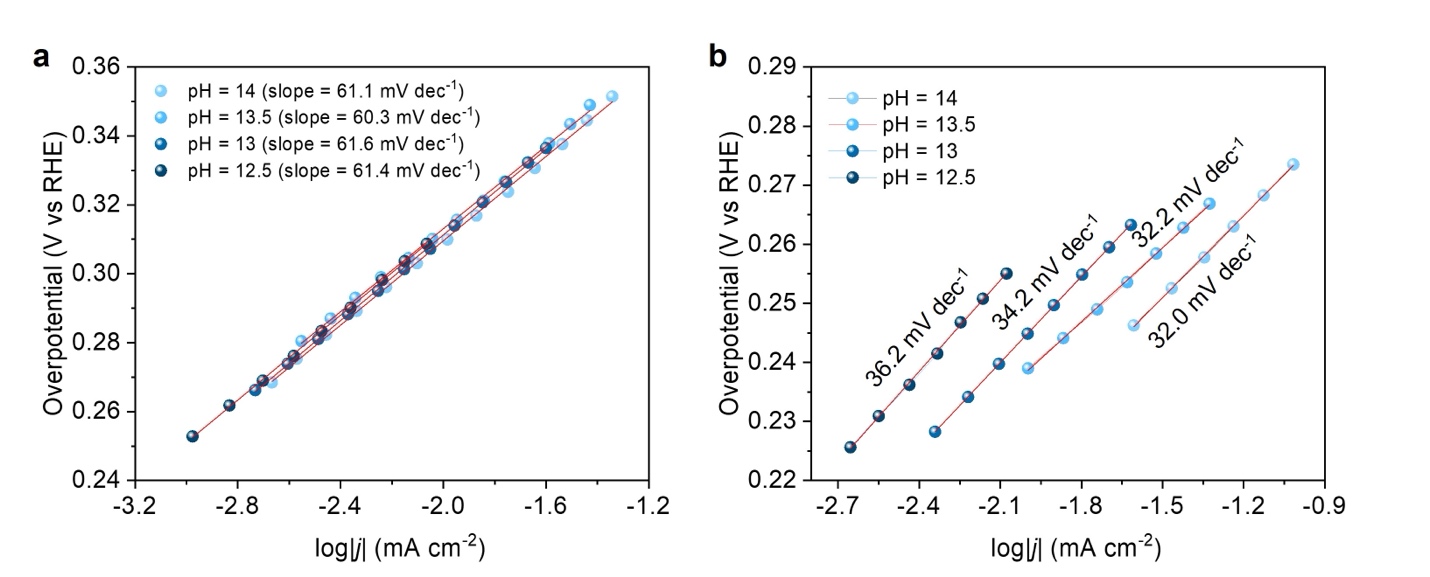


**Figure S19.** Tafel plots as a function of electrolyte pH for (a) Ni_3_N/NF and (b) FeOOH@Ni_3_N/NF.

**References**

1. [] P. Juhás, T. Davis, C.L. Farrow, S.J.L. Billinge, *J. Appl. Crystallogr.* **2013**, *46*, 560–566. [↑](#endnote-ref-1)
2. [] S. Narayanaru, H. Kuroki, T. Tamaki, G.M. Anilkumar, T. Yamaguchi, ***RSC Sustainability*** **2025**, ***3***, 1705–1713. [↑](#endnote-ref-2)
